# Importance of MAP Kinases during Protoperithecial Morphogenesis in *Neurospora crassa*

**DOI:** 10.1371/journal.pone.0042565

**Published:** 2012-08-10

**Authors:** Alexander Lichius, Kathryn M. Lord, Chris E. Jeffree, Radek Oborny, Patid Boonyarungsrit, Nick D. Read

**Affiliations:** 1 Fungal Cell Biology Group, Institute of Cell Biology, The University of Edinburgh, Edinburgh, United Kingdom; 2 Institute of Molecular Plant Sciences, The University of Edinburgh, Edinburgh, United Kingdom; University of Nebraska, United States of America

## Abstract

In order to produce multicellular structures filamentous fungi combine various morphogenetic programs that are fundamentally different from those used by plants and animals. The perithecium, the female sexual fruitbody of *Neurospora crassa*, differentiates from the vegetative mycelium in distinct morphological stages, and represents one of the more complex multicellular structures produced by fungi. In this study we defined the stages of protoperithecial morphogenesis in the *N. crassa* wild type in greater detail than has previously been described; compared protoperithecial morphogenesis in gene-deletion mutants of all nine mitogen-activated protein (MAP) kinases conserved in *N. crassa*; confirmed that all three MAP kinase cascades are required for sexual development; and showed that the three different cascades each have distinctly different functions during this process. However, only MAP kinases equivalent to the budding yeast pheromone response and cell wall integrity pathways, but not the osmoregulatory pathway, were essential for vegetative cell fusion. Evidence was obtained for MAP kinase signaling cascades performing roles in extracellular matrix deposition, hyphal adhesion, and envelopment during the construction of fertilizable protoperithecia.

## Introduction

The perithecium is the female sexual reproductive organ, or fruitbody, of *Neurospora crassa* within which *ascospores*, the products of meiosis, are generated [1], [2]. The perithecium is composed of at least 14 morphologically distinct cell-types [2], [3], and is formed by various processes including: hyphal aggregation; adhesion; septation; branching; and cell differentiation. As a result of these processes, filamentous fungi achieve multicellularity in a way that is fundamentally different from that in plants or animals, with the important point being that fungal tissues and organs are formed from the growth, aggregation and differentiation of hyphae [2], [4]. Fruitbody morphogenesis in *N. crassa* provides an excellent model system for the study of fungal multicellular development. Perithecium morphogenesis in *N. crassa* and other members of the Sordariomycetes (e.g. *Sordaria* spp., *Podospora* spp., *Gelasinospora* spp. and *Chaetomium* spp.) has three main stages (ascogonial, protoperithecial and perithecial), each involving the differentiation of several morphologically distinct cell-types [2], [3]. The ascogonial stage of development is observed as a very small (5–20 µm in diameter) coiled hyphal branch, also described in *N. crassa* as a hyphal knot [2], [3]. The protoperithecial stage is initiated by *enveloping hyphae* [3] that wrap around the *ascogonium* [3] to form an almost spherical, or more specifically subspherical, structure [5]. Initiation of the protoperithecial stage represents a key morphogenetic event, when differentiation of distinct, multicellular tissues commences [2]. Fruitbody expansion and internal differentiation lead to the mature ‘female’ protoperithecium. Protoperithecia of *N. crassa* can form one or more *trichogynes*. Each trichogyne is a specialized ‘female’ hypha that is required for non-self fusion with cells of the opposite mating-type. Trichogynes can grow to several hundred micrometers in length [6] and can form branches [7]. The peptide sex-pheromone [8], [9] released by the ‘male’ triggers the homing response of ‘female’ trichogynes [6], [7]. The fertilizing agent (spermatium) may be: an asexual spore, the *conidium*, of which there are three types in *N. crassa* (*macroconidia*, *microconidia* and *arthroconidia* [3]); a germinated ascospore (meiospore); or indeed any vegetative cell or hypha of the mating partner [10], [11]. In *heterothallic* species, such as *N. crassa*, fertilization by an opposite mating-type ‘male’ partner provides the necessary signal for the transition from the protoperithecial to the perithecial stage of fruitbody development [12]. In *homothallic*, self-fertile Sordariomycetes such as *Sordaria macrospora*, that lack any type of conidium or trichogyne, mating and hence, non-self fusion is not a requirement for progression to the perithecial stage [2], [12], [13], [14], [15]. However, certain members of the Sordariomycetes possess asci with four ascospores and each ascospore contains nuclei of opposite mating-type. As a result, each ascospore behaves as if it were homothallic; this condition is termed secondary homothallism (or pseudohomothallism) [16], [17], [18]. In secondary homothallic species, such as *N. tetrasperma*, mating involving trichogyne and spermatium fusion does not commonly occur [16], [17], [18], [19].

Upon successful mating-cell fusion (plasmogamy) in *N. crassa*, the male nucleus travels through the trichogyne into the ascogonium [3] of the yet unfertilized protoperithecium. Arrival of the ‘male’ nucleus inside the ascogonium induces continued differentiation and further expansion of the protoperithecium. It also initiates the dikaryotic phase of the life cycle, which is restricted to the developing *ascogenous hyphae* [3] within the differentiating perithecium [11], [20]. Increasing melanization of the perithecial wall cells ultimately leading to the almost-black mature perithecium, is a visual marker of continued sexual development upon fertilization [21]. In *N. crassa*, dikaryotic cells are generated and maintained by the formation of specialized hyphal compartments called *croziers* [22]. Recurrent cycles of crozier cell fusion generate binucleate cells subtending the tip cell of ascogenous hyphae [1], [22], [23], inside which subsequent nuclear fusion (karyogamy) yields a very short-lived diploid stage prior to meiosis, ascospore development and ascospore delimitation [24], [25]. Whether crozier fusion can be considered a non-self fusion or a self-fusion process, i.e. regulated by genes expressed from both parent nuclei or from only one, remains unresolved.

Mitogen-activated protein (MAP) kinase phosphorylation cascades are highly conserved and well characterized signaling pathways in eukaryotes [26], [27], [28]. In *Saccharomyces cerevisiae*, a total of 16 MAP kinases constitute five partially overlapping signaling pathways that are involved in regulating pheromone-induced mating, filamentous growth, cell wall modification and repair, responses to high osmolarity and ascospore wall assembly [26], [29], [30], [31], [32], [33]. Three-tiered MAP kinase modules comprising orthologs to nine of the budding yeast MAP kinases, have been identified in *N. crassa* [34], [35] and are regarded to be equivalent to the pheromone response (PR) pathway, the cell wall integrity (CWI) pathway, and the osmoregulatory (OS) pathway from budding yeast [26], [32]. Gene-deletion mutants of the PR-MAP kinase pathway were amongst the first hyphal-fusion mutants characterized in *N. crassa* [36], [37], [38], [39], and connections between their pleiotropic phenotype and defects in fruitbody morphogenesis have been previously recognized [23], [40], [41]. A model summarizing all nine MAP kinase components and their functions in *N. crassa*, including the cross-communication with other signaling pathways involved in filamentous growth and sexual morphogenesis, has recently been provided [41]. Hitherto, studies have documented that all nine MAP kinase mutants are unable to differentiate fertilizable perithecia, but the specific stages of development at which defects occur have remained mostly uncharacterized. Reports of defective sexual fruitbody development resulting from MAP kinase mutations have been made in *Magnaporthe grisea* [42], *Fusarium graminearum* [43], *Podospora anserina* [44], [45], [46], *Cochliobolus heterosporus* [47], and *Aspergillus nidulans* [48], [49], but overall, detailed ultrastructural studies of these defects are lacking. Furthermore, neither has a direct connection between hyphal fusion and fruitbody morphogenesis been established.

This study, firstly analyzed the key morphogenetic stages comprising protoperithecial development in the *N. crassa* wild-type in greater detail than has been accomplished so far, secondly addressed the specific role of MAP kinases in this process, and thirdly asked the question: to what extent do defects in vegetative hyphal fusion (VHF) influence protoperithecial morphogenesis?

## Materials and Methods

### Media and culture conditions

Strains were maintained on solid (2% agar) or in liquid Vogel's minimal medium (VMM) [50] with 2% sucrose using standard *N. crassa* cultivation techniques [11]. For Ignite selection (*bar* resistance gene), NH_4_NO_3_ was substituted by 0.5% (w/v) proline as alternative nitrogen source to increase the potency of Ignite selection at an effective final concentration of 400 µg/ml [51]. For hygromycin B selection (*hph* resistance gene) [52] or nourseothricin selection (*nat1* resistance gene), [53] drugs were added at final concentrations of 200 µg/ml and 30 µg/ml, respectively. To induce the sexual cycle in *N. crassa*, strains were grown under nitrogen- and carbon-limiting conditions on solid synthetic crossing medium (SCM) [54] and low-sucrose (0.2% sucrose in dH_2_O) agar (LSA), in most cases overlaid with cellophane. Development of conidial germlings, including the quantification of conidial anastomosis tube (CAT)-mediated cell fusion, was assessed as described in detail previously [55], [56].

### Selection of gene-deletion mutants for morphogenetic analysis

Previous work on sexual development in *N. crassa* has used mutant strains generated by different methods and obtained from a variety of sources (references in Table 1). Therefore, we decided to verify earlier findings using gene-deletion strains (*a.k.a.* gene knock-out (KO) strain) exclusively generated by homologous recombination and obtained only from one source, in this case the Fungal Genetics Stock Center (FGSC, Kansas City, Missouri, USA) [57], [58]. Our analysis included new gene-deletion strains that, due to updated annotation of the *N. crassa* genome or problems noted by the community with older strains, recently became available. Replacement strains used in this study were Δ*nrc-1* FGSC18162, Δ*os-4* FGSC18202, Δ*os-5* FGSC18203, and Δ*os-2* FGSC17933. Homokaryons of Δ*nrc-1* FGSC18162 were generated through single spore propagation of asexual conidia (micro- and macroconidia) on hygromycin B selection medium.

**Table 1:** Stages of fruitbody development accomplished by *N. crassa* cell-fusion mutants used as females in heterozygous crosses with the wild type.

References	this study, [41], [89], [90]	this study, [41], [89], [90]	this study, [41], [89], [90]	this study, [40], [41]	this study, [40], [41]	this study, [40], [41]	this study, [36], [41]	this study, [41]	this study, [36], [37], [41]
Ascospore germination									
Ascospore ejection									
Neck/ostiole development									
Ascospore production/maturation									
Ascogenous hyphae									
Crozier fusion									
Perithecium differentiation									
Nuclear transit/fertilization									
Trichogyne homing and fusion									
Fertilizable protoperithecium									
Trichogyne emergence									
Fruitbody expansion							**√**	**√**	**√**
ECM deposition/hyphal adhesion							**√**	**√**	**√**
Enveloping hyphae				**√**	**√**	**√**	**√**	**√**	**√**
Septation/branching				**√**	**√**	**√**	**√**	**√**	**√**
Ascogonial coil formation				**√**	**√**	**√**	**√**	**√**	**√**
**Protein**	**MAP3K**	**MAP2K**	**MAPK**	**MAP3K**	**MAP2K**	**MAPK**	**MAP3K**	**MAP2K**	**MAPK**
**FGSC strainnumberLocus number**	**FGSC18202NCU03071**	**FGSC18203NCU00587**	**FGSC17933NCU07024**	**FGSC11326FGSC11327NCU02234**	**FGSC11318FGSC11319NCU06419**	**FGSC11320FGSC11321NCU09842**	**FGSC11466NCU06182**	**FGSC11524NCU04612**	**FGSC11482NCU02393**
**Gene**	***os-4***	***os-5***	***os-2***	***mik-1***	***mek-1***	***mak-1***	***nrc-1***	***mek-2***	***mak-2***

Tick marks indicate completed stages of sexual developmental. Empty boxes show developmental stages identified as being fully blocked. (MAP3K, MAP kinase kinase kinase; MAP2K, MAP kinase kinase; MAPK, MAP kinase).

### Polymerase chain reaction (PCR) genotyping of *N. crassa* gene-deletion mutants

All KO strains used in this study (Table 2) were produced and verified by Southern blotting within the NIH *Neurospora* Genome Knock-Out Project [59]. The genotype of the deposited strains can be looked up in the regularly updated master spreadsheet of the *Neurospora* Genome Project: http://www.dartmouth.edu/~neurosporagenome/knockouts_completed.html. Additionally, the replacement of targeted open reading frames (ORF) by the *hph*-knock-out cassette was verified for each strain within this study. For this, genomic DNA was purified after phenol/chloroform extraction and analyzed by PCR (Figure S1 and S2). Specific primer pairs were used to probe for: (1) the absence of the target gene from its original locus; (2) the presence of the *hph*-KO cassette at this locus; (3) the absence of the target ORF from the whole genome; and (4) the presence or absence of *mus51* and *mus52* loci that could indicate whether the obtained gene-deletion strain had been successfully recovered after back-crossing to the wild type. When reactions were performed as multiplex PCRs [60], an additional pair of oligonucleotides binding within the actin locus (NCU04173.3) was used as an internal positive control for each reaction. Table S1 lists all primers used for genotyping in this study.

**Table 2:** *N. crassa* strains used in this study.

*Strain*	*FGSC/strain number*	*Locus/Host strain*	*Mating type*	*Genotype*
*wild type*	FGSC2489	_	*A*	*74-OR23-1VA*
*wild type*	FGSC4200	_	*a*	*ORS-SL6a*
*Δmek-1*	FGSC11318	NCU06419.2	a*	*Δmek-1::hygR*
*Δmek-1*	FGSC11319	NCU06419.2	A*	*Δmek-1::hygR*
*Δmak-1*	FGSC11320	NCU09842.1	A*	*Δmak-1::hygR*
*Δmak-1*	FGSC11321	NCU09842.1	a*	*Δmak-1::hygR*
*Δmik-1*	FGSC11326	NCU02234.2	A*	*Δmik-1::hygR*
*Δmik-1*	FGSC11327	NCU02234.2	a*	*Δmik-1::hygR*
*Δos-2*	FGSC11436	NCU07024.2	A*	*Δos-2::hygR*
*Δnrc-1*	FGSC11466	NCU06182.2	a*	*Δnrc-1::hygR, Δmus51::bar^+^*
*Δmak-2*	FGSC11482	NCU02393.2	a*	*Δmak-2::hygR, Δmus51::bar^+^*
*Δmek-2*	FGSC11524	NCU04612.2	a*	*Δmek-2::hygR, Δmus51::bar^+^*
*Δos-2*	FGSC17933	NCU07024.2	A*	*Δos-2::hygR*
*Δnrc-1*	FGSC18162	NCU06182.2	*a (het)*	*Δnrc-1::hygR, Δmus51::bar^+^*
*Δnrc-1*	this study	NCU06182.2	*a* HS*	*Δnrc-1::hygR, Δmus51::bar^+^*
*Δos-4*	FGSC18202	NCU03071.2	a*	*Δos-4::hygR, Δmus51::bar^+^*
*Δos-5*	FGSC18203	NCU00587.2	a*	*Δos-5::hygR, Δmus51::bar^+^*
*wt MAK-1-sGFP*	NCAL007	FGSC4200	*a*	*wt::Pccg1::mak-1-sgfp::bar+*
*Δmak-1 MAK-1-sGFP*	NCAL010	FGSC11320	*A*	*Δmak-1::hygR; Pccg1::mak-1-sgfp::bar+*
*wt OS-2-sGFP*	NCAL016	FGSC2489	*A*	*wt::Pccg1::os-2-sgfp::bar+*
*Δos-2 OS-2-sGFP*	NCAL018	FGSC11436	*A*	*Δos-2::hygR; Pccg1::os-2-sgfp::bar+*
*Δos-2 OS-2-sGFP*	NCAL020	FGSC17933	*A*	*Δos-2::hygR; Pccg1::os-2-gfp::bar+*
*wt MAK-2-sGFP*	NCAL037	FGSC4200	*a*	*wt::Pccg1::mak-2-sgfp::nat1*
*Δmak-2 MAK-2-sGFP*	NCAL043	FGSC11482	*a*	*Δmak-2::hygR; Pccg1::mak-2-sgfp::nat1*

Asterisks denote strains that were genotyped by PCR; HS (homokaryon selection): denotes strains of which homokaryons were generated by repeated isolation of monosporic microcolonies on selection medium.

### Assessment of female and male fertility

To evaluate female fertility, gene-deletion strains were inoculated onto SCM or LSA plates and incubated for 2–4 days at 25°C in constant light. In parallel, a wild type strain of opposite mating-type was cultured on standard VMM for 2–3 days at 30°C until sufficient conidia had developed. Protoperithecia, which usually developed after 3–4 days on the KO mycelium, were fertilized with opposite mating-type ‘male’ conidia from the wild type, either ‘dry’ or ‘wet’. For dry-fertilization, male conidia were collected on the Petri dish lid, by inverting the culture, and subsequently transferred onto the female mycelium by exchanging the lid onto the female culture plate and gently tapping off the male conidia. For wet-fertilization, male conidia were harvested in sterile water and either evenly distributed onto the female mycelium by flooding, or applied as 5 µl droplets at defined positions. The same procedure was employed to test male fertility, except that the wild type was used as female partner and conidia of the opposite mating-type KO mutant were used as the ‘male’. Confrontation crosses, whereby either of the two parental strains may act as male or female partner, were performed by co-inoculating the KO mutant strain with a wild type strain of opposite mating-type on the same SCM or LSA plate, followed by incubation at 25°C over the next 2–3 weeks.

In all crosses, development of protoperithecia and the differentiation into mature perithecia was monitored under a stereomicroscope for three weeks post-fertilization. The appearance of ascospores was defined as the determining feature of successful sexual reproduction. Ascospores were collected from the Petri dish lid, microscopically analyzed and then cultured on selection medium to assess viability. In crosses where perithecia appeared within that time period, but no ascospores could be recovered, the perithecia were cracked open, using dissecting needles, to evaluate ascus and ascospore development.

### Evaluation of osmosensitive MAP kinase mutants

In order to phenotypically verify putative OS-MAP kinase mutants, fungal development was tested under salt stress (3% and 6% w/v NaCl) and in the presence of the phenylpyrrol fungicide fenpiclonil (1.5 or 4.5 µM). Conidial germling assays were performed as described earlier [55]. Radial colony extension rates were assessed on VMM agar plates, and if required, supplemented with salt or fungicide as described above. For this, inoculated plates were incubated overnight (∼16 h) at 25°C. The next morning, margins of four randomly chosen radii were marked in each colony. In some cases, the plates were then transferred to 35°C and extension of the colony edges was marked every 2 h for a period of 8 h. In all cases, maximal extensions rates were measured and mean extension rates of duplicate samples were calculated (n = 8 for each tested condition).

### Plasmid construction

The MAK-1-sGFP expression plasmid pAL1-MAK-1 was constructed by first generating the GFP expression vector pAL1 through subcloning the sGFP coding region from pMF272 [61] into pBARGRG1 [62] using *Bam*HI/*Eco*RI restriction/ligation, and subsequently ligating the *mak-1* gene amplified from *N. crassa* wild type cDNA using oligonucleotides mak1_*BamH*I_fw 5′-GATCGGATCCATTCGCCATGGCTGATCTCGTG-3′ and mak1_*Xma*I_rv 5′-GATCCCCGGGATTCGCCATGGCTGATCTCGTG-3′ (*BamH*I and *Xma*I restriction sites underlined) into *Bam*HI/*Xma*I linearized pAL1 in-frame to sGFP. Plasmid pAL1-OS-2 for the expression of OS-2-sGFP was generated by amplifying the coding region for *os-2* from *N. crassa* wt cDNA using oligonucleotides os2_if_*BamH*I_fw 5′-TTTCCTCGACGGATCCATGGCCGAATTTATCCGC-3′ and os2_GS_if_rv 5′-AGACACCATCGAGCCTTGCGGCGGAACATCTTC-3′ (underlined are the 15 bp overlaps required for recombination), then amplifying the sGFP coding region from pAL1-MAK-1 using oligonucleotides GS_sGFP_if_fw 5′-CCGCCGCAAGGCTCGATGGTGAGCAAGGGCGAGG-3′ and sGFP_if_EcoRV_rv 5′-ATCGATAAGCTTGATATCTTACTTGTACAGCTCGTCCATGCC-3′, and subsequently joining both purified PCR products with *BamH*I/*EcoR*V-linearized and gel-purified pBARGRG1 using In-Fusion® PCR cloning (Clontech, UK). The same technique was used to recombine the PCR products of the *mak-2* ORF amplified from wt cDNA using oligonucleotides mak2_if_*Bam*HI_fw 5′-TTTCCTCGACGGATCCATGAGCAGCGCACAAAGAGG-3′and mak2_GS_GFP_if_rv 5′-AGACACCATCGAGCCCCTCATAATCTCCTGGTAGATCAACTGC-3′, and the coding region of GFP amplified from pAL1-MAK-1 using oligonucleotides mak2_GS_GFP_if_fw 5′-ATTATGAGGGGCTCGATGGTGTCTAAGGGCGAAGAGC-3′and GFP_if_EcoRV_rv 5′-TCGATAAGCTTGATATCTTACTTGTACAGCTCGTCCATGC-3′, into *Bam*HI/*Eco*RV linearized and gel-purified pAL4-Lifeact [63], in order to generate pAL7-MAK-2, the expression plasmid for MAK-2-GFP. Upon propagation through *E. coli*, recovered plasmids were verified by sequencing and transformed into *N. crassa* wild type, Δ*mak-1*, Δ*os-2* and Δ*mak-2* strains, respectively (Table 2). Expression of all GFP fusion constructs was under control of the glucose-repressible *Pccg-1* promoter [61], [64].

### Transformation and transformant selection

Transformations were performed using a standard electroporation protocol for *N. crassa* as described previously [65]. MAK-1-GFP, OS-2-GFP and MAK-2-GFP expressing strains were created by random integration of pAL1-MAK-1, pAL1-OS-2 and pAL7-MAK-2, respectively, into the genomes of wild type strains FGSC4200 (*mat a*), FGSC2489 (*mat A*), and gene-deletion mutant strains Δ*mak-1* (FGSC11320), Δ*os-2* (FGSC11436 and FGSC17933), and Δ*mak-2* (FGSC11482), respectively (Table 2). Transformants were selected by recovery on either nitrogen-free selection medium containing Ignite (pAL1-MAK-1 and pAL1-OS-2) or standard selection medium containing nourseothricin (pAL7-MAK-2), and by expression of the fluorescent fusion construct in conidial germlings using light microscopy. Furthermore, phenotypic rescue of the transformed gene-deletion strains served as the most reliable marker for successful integration of a functional copy of the MAP kinase-GFP fusion protein into the genome.

### Stereomicroscopy

A Nikon SMZ 1500 fluorescence stereomicroscope (Nikon Instruments Europe BV, UK), with a magnification range of 0.75× to 11.25×, and a mercury arc lamp excitation light-source were used to assess general colony morphology, monitor development of sexual structures, and to evaluate expression of fluorescent fusion proteins within transformant strains. GFP was visualized with a GFP (excitation 470/40 nm; 505 nm LP dichroic mirror; emission 530/40 nm) filter set. Images were acquired with Nikon ACT-1 software on a Nikon digital DXM 1200F color camera and stored as uncompressed tagged-image file format (TIFF).

### Widefield fluorescence and differential interference contrast (DIC) microscopy

For DIC microscopy, an inverted Nikon TE2000-U Eclipse widefield microscope (Nikon Instruments Europe BV, UK) equipped with Wollaston polarizer, prism and analyzer was used, along with a Nikon Plan Fluor 100×/1.4 N.A. DIC H oil immersion, Nikon Plan Apo 60×/1.2 N.A. DIC H water immersion, and Nikon Plan Fluor 20×/0.5 N.A. dry objectives fitted with the corresponding DIC lens sliders. Images were acquired with Nikon ACT-1 software on a Nikon digital DXM1200F color camera and stored as TIFF. For widefield fluorescence microscopy, the same microscope and objectives were used with: a CoolLED *p*E-2 excitation system (http://www.coolled.com); 470 nm LED array module with a Nikon B-2A filter for GFP imaging, and a 380 nm LED array module with a Nikon UV-2A for Calcofluor White (CFW) imaging. Image capture was with a Hamamatsu Orca-ER C4742-80 camera (Hamamatsu Photonics UK Ltd, Welwyn Garden City, UK) and MetaMorph software v7.7.6.0 (Molecular Devices LLC, Sunnyvale CA, USA, http://www.moleculardevices.com). Samples were prepared using the inverted-agar-block method [66] and CFW staining was as previously described [67]. Optical sectioning was performed with a P-721 PIFOC Z objective focusing system connected to an E-625 PZT piezo servo controller (www.physikinstrumente.com) allowing rapid z-stack acquisition with 0.2 to 0.5 µm step size. Apart from basic brightness, contrast and display range adjustments using the ImageJ freeware platform (rsbweb.nih.gov/ij/) no further manipulation, such as deconvolution, were used to prepare the raw data for presentation.

### Low-temperature scanning electron microscopy (LTSEM)

All samples for LTSEM were prepared and incubated in the same way as for other applications described previously [38], either on VMM agar plates (mature hyphal colonies and conidial germlings) or SCM or LSA plates (development of protoperithecia) overlaid with sterile cellophane (525 gauge uncoated Rayophane, A.A. Packaging, Preston, UK) to allow rapid sample preparation. At desired time points ∼12 mm^2^ cellophane rectangles carrying the specimen were cut out, adhered to the cryospecimen carrier (Gatan, Oxford, UK) with Tissue-Tek OCT compound (Sakura Finetek, Torrance, USA) then immediately cryofixed by plunging into subcooled liquid nitrogen. The specimen carrier was transferred under low vacuum to the cold stage (−120°C) of a 4700II field-emission scanning electron microscope (Hitachi, Wokingham, UK). On the stage the samples were partially freeze-dried at −80°C to remove surface ice by sublimation; cooled down to −120°C; sputter-coated in a Gatan Alto 2500 cryopreparation system at −180°C and coated with ∼10 nm of 60∶40 gold-palladium alloy (Testbourne Ltd., Basingstoke, UK) in an argon gas atmosphere. The specimen was examined at −160°C with a beam accelerating voltage of 2 kV, a beam current of 10 µA, and working distances of 12–15 mm. Digital images were captured at a resolution of 2560×1920 pixels using in most cases the signal from the lower secondary electron detector, and saved as TIFF.

## Results

### Protoperithecial development in the *N. crassa* wild type

#### Ascogonial coil formation

**Figure 1:**
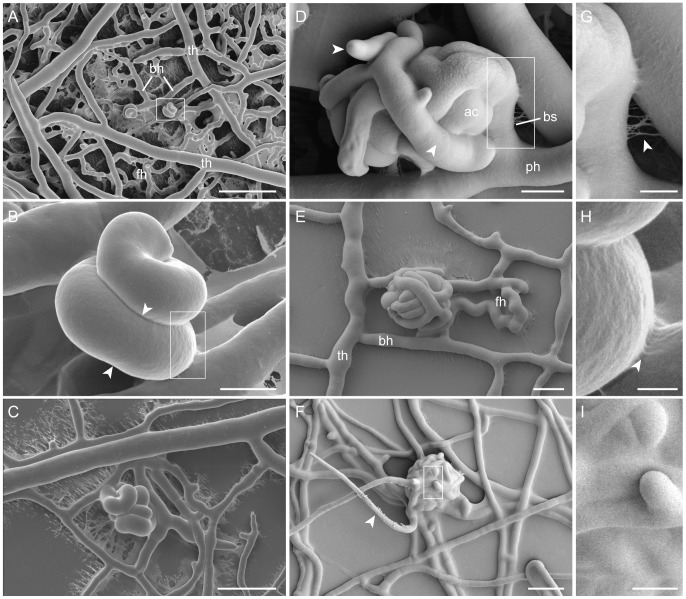
Protoperithecial morphogenesis of *N. crassa* wild type. LTSEM of the main stages of protoperithecial development. (**A**) Two ascogonial coils differentiated from the vegetative mycelium of a two day-old culture. These two coils have formed on branches (*bh*) off the main arterial trunk hyphae (*th*). Some of the surrounding branches have fused with each other, they are therefore considered to be fusion hyphae (*fh*). Vegetative hyphal fusion is instrumental in the establishment of a fully co-operative interconnected mycelium. Scale bar, 50 µm. (**B**) Higher magnification of the ascogonial coil boxed in (A). On careful inspection a septum can be seen on the lower part of the coil (aligned with arrowheads). Scale bar, 5 µm. (**C**) A slightly expanded ascogonial coil again formed on a side branch of a trunk hypha, the coil is being wrapped around by enveloping hyphae. Scale bar, 20 µm. (**D**) A slightly later stage where enveloping hyphae (arrowheads) originating from the ascogonium have wrapped around the central ascogonial coil (*ac*). These enveloping hyphae exhibit septation and branching. The ‘parent hypha’ (*ph*) of the ascogonial coil can be clearly defined, and is separated from the developing fruitbody by a basal septum (*bs*). (**E**) The subspherical shape of the protoperithecium becomes evident after additional enveloping hyphae have formed a protective casing around the ascogonium. Trunk hyphae (*th*), their branches (*bh*) and fusion hyphae (*fh*) can be clearly distinguished. Scale bar, 5 µm. (**F**) Mature protoperithecium, with visible ECM secretion ‘gluing’ enveloping hyphae together, and a trichogyne (arrowhead) emerging from its center. Scale bar, 20 µm. (**G**) Enlarged view of the boxed area in (D) showing ECM strands between hyphae (arrowhead). Scale bar, 2 µm. (**H**) Enlarged view of the boxed area in (B) showing ECM strands (arrowhead) between the tightly attaching revolutions of the ascogonial coil. Scale bar, 1 µm. (**I**) Enlarged view of the boxed area in (F) showing the surface hyphae of the protoperithecium evenly covered in ECM. Scale bar, 5 µm.

**Figure 2:**
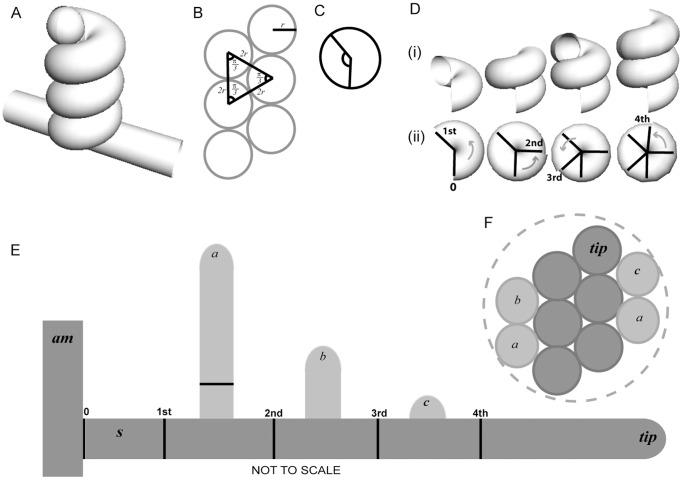
Simulation of the transition from two-dimensional hyphal growth into a three-dimensional helical object representing the ascogonial coil. (**A**) Mathematically drawn model of an ascogonial coil, helix (*cos t, sin t, t*) from *t = 0 to 6π* (3 full circles). The ascogonial mother-cell is shown as a cylinder and the hyphal tip of the coiling branch is represented as a hemisphere. (**B**) A vertical cross-section through the coil shown in (A) where the distance between the centers of each circular cross-section is equal to the hyphal diameter (2*r*). (**C**) Diagram indicating the angle 2.4 *rad* (∼137.5°). (**D**) Position of septa from microscopical observations of numerous ascogonial coils in *N. crassa*. Septa are usually observed around two thirds of a revolution (240°) apart, after the coil-tip has made more than one complete revolution (2*π rad* or 360°). The angle between projected septa is likely to be optimized around 2.4 *rad* for maximum structural strength and this is represented here in the cut-away sections (i) of the coil shown in (A). (ii) The positions of the subsequent septa are shown in top view. The angle between ‘septa’ approximates to 2π–2.4 *rad* (∼222.5°). (**E**) Diagrammatic representation of an unwrapped-coil (not to scale), showing septation (black vertical lines) and branching of successive enveloping hyphae: (a), (b), and (c) (paler grey) of the coil. Branching is assumed here to occur equidistant between septa, although, *in vivo* the branching sometimes appears nearer to one septum. A stalk-cell is often observed *in vivo*. The diagram illustrates this with a basal septum (0) making a ‘stalk-cell’ compartment (s) next to the ascogonial mother-cell (am). (**F**) Extrapolated representation of a vertical cross-section of a simulated ascogonial coil, which has been wrapped by enveloping hyphae that would have originated from the septated compartments shown in (E). Note that the resulting coiled structure (not to scale) is approaching that of a sphere (represented by the dashed outer-circle). *N.B. In vivo*, enveloping hyphae tend to be narrower in diameter than the ascogonial mother-cell.

An ascogonial coil initial differentiates as a branch from a compartment of a vegetative hypha in the sub-peripheral region of the colony. It usually emerges from a *trunk hypha* (the thickest hyphae of the sub-peripheral mycelium) [3] or one of its branches with a thickness of 5–10 µm (Figure 1A). This ascogonial branch, with similar hyphal diameter to its ‘*parent hypha*’, immediately coils around on itself and adheres to itself to form a tight helical structure (Figure 1B), where the pitch of the helix is equivalent to the width of the coiling hypha (Figures 1B, 2A and 2B). This is very similar to observations made in *S. macrospora* [2] and *S. humana* [68]. After the tip of the ascogonial coil had made a complete revolution, *septa* (dividing cross-walls) were being formed in the earlier part of the coil (arrowheads in Figure 1B). These septa were positioned approximately two-thirds of a revolution of the helix apart from the previous septa (Figures 2C and 2D). Branches emerged from the septated ascogonial compartments, but not from the ascogonial-tip cell (Figures 1C and 2E). These branches, in turn, enveloped the coil, ‘hugging’ its surface whilst following the grooves formed on the surface of the ascogonial coil between adjoining hyphae (Figures 1D and 2F). These enveloping hyphae, continue to septate, branch, and further wrap around the ascogonial-coil core, referred thereafter as the *ascogonium*. Enveloping hyphae are often narrower (<5 µm width) than the ascogonial-coil hypha. Residues of extracellular matrix (ECM) secretion, presumably required for the tight adhesion between the revolutions of the ascogonial coil to itself and between the coil and enveloping hyphae, are shown in enlarged views in Figures 1G and 1H.

#### Protoperithecium expansion

After enveloping hyphae which originated from the ascogonial compartments have enlarged the structure to a diameter of 20–30 µm, additional enveloping hyphae emerge, either as branches of the initial enveloping hyphae (Figure 1D) or from neighboring vegetative hyphae and envelop the young fruitbody further. ECM accumulates on the outer surface of the forming protoperithecium at this early stage of development, and eventually covers the whole structure evenly (Figure 1I). Usually, after the structure has expanded to a diameter of 40–50 µm and enveloping hyphae have established a compact casing around the ascogonium, the product of fruitbody expansion is a subspherical protoperithecium (Figure 1E).

#### Trichogyne emergence

**Figure 3:**
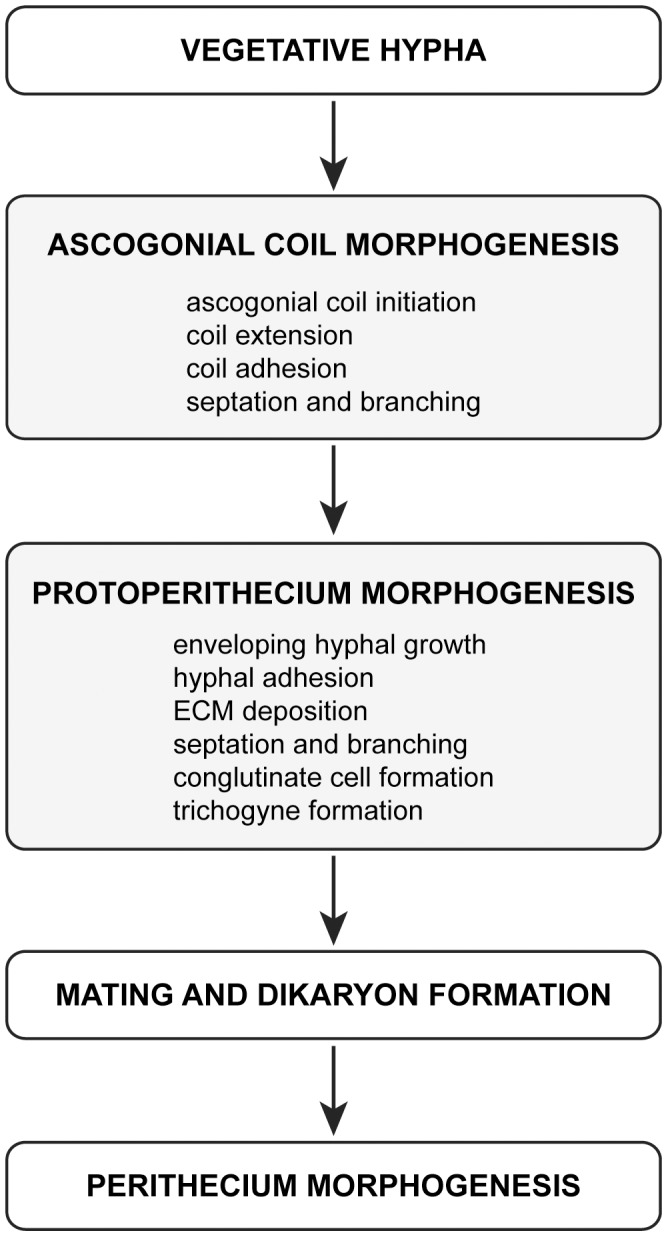
Main stages of protoperithecial development. The ascogonium forms as a specialized, coiled, hyphal branch from a ‘parent hypha’ of the vegetative mycelium. The coil expands, adheres to itself, septates and branches. It sends out more branches, which envelop it. Additional, enveloping hyphae from neighboring areas of the vegetative mycelium, aggregate, reinforce and expand the protective casing around the ascogonium. Secretion of ECM is a precursor to hyphal adhesion during this process, which potentially also involves hyphal fusion. Continued fruitbody expansion, cellular differentiation through septation, branching and cell conglutination (*conglutinate cells* are those that have adhered to each other), melanization and emergence of the trichogyne mark the final stages of protoperithecium maturation. Mating-cell fusion leading to fertilization and dikaryon formation mark the transition into perithecial development. Autonomous developmental stages of the protoperithecium are highlighted with grey shading. Table 3 summarizes the range of sizes observed for these main developmental stages observed during protoperithecium morphogenesis.

Protoperithecial expansion in *N. crassa* usually arrests when the fruitbody has reached a diameter of 80–100 µm. At this stage, elongated trichogynes will have emerged (arrowhead in Figure 1F) resembling the endpoint of female-autonomous protoperithecium maturation. For the transition into perithecium morphogenesis, non-self, mating cell-fusion is required. Figure 3 summarizes the main stages of sexual fruitbody development in *N. crassa* schematically. Table 3 provides an overview of the range of sizes observed throughout these main morphogenetic stages.

**Table 3:** Size ranges of developmental stages during protoperithecium morphogenesis.

Developmental stage	Min. ø	Max. ø	Mean ø *
Trunk hypha	5 µm	10 µm	6 µm
Ascogonial coil	9 µm	13 µm	12 µm
Ascogonial coil with branches and enveloping hyphae	10 µm	24 µm	17 µm
Subspherical protoperithecium with enveloping hyphae	24 µm	100 µm	60 µm
Mature protoperithecium	37 µm	100 µm	74 µm

* Mean diameter were calculated from 100 individual measurements on 2–6 day old wild type cultures grown on LSA.

### Genotypic verification of MAP kinase gene-deletion mutants

#### PCR-based genotyping confirmed targeted gene deletion in all MAP kinase mutants

PCR genotyping confirmed that the targeted open reading frames have been successfully exchanged for the *hph*-gene deletion cassette in all MAP kinase KO mutants used in this study (Figure S1 and S2). Residual presence of wild type genes indicated weak heterokaryotic background for three of the nine strains, however, this can be regarded as insignificant as it did not alter the dominant mutant phenotypes of these strains (for additional information please refer to Figure S1 and S2 legends.)

#### MAP kinase mutant phenotypes co-segregated with hygromycin B resistance

PR- and CWI-MAP kinase gene-deletion strains identical to those used in this study have previously been verified as being correct by back-crossing and co-segregation analyses [69]. Very recently, the same analysis has been conducted for all OS-MAP kinase mutants available from the FGSC by the same group, showing significant co-segregation of the mutant phenotype with the hygromycin B-resistance marker in the evaluated progeny of the ‘new’ *os* mutants: Δ*os-4* FGSC18202 (91%), Δ*os-5* FGSC18203 (95%) and Δ*os-2* FGSC17933 (100%) (ratio of co-segregation indicated as percentages). In contrast, the same mutant phenotype did not co-segregate in any of the ‘old’ (Δ*os-4*, FGSC11479; Δ*os-5*, FGSC11480 and Δ*os-2*, FGSC11436) OS-MAP kinase mutant progeny (S. Free, pers. comm.).

This data, taken together with our own PCR-based genotyping analyses (previous section) and the consistency of the mutant phenotypes within and between the MAP kinase cascades (see following sections), provide very strong evidence that the developmental phenotypes described in this study are exclusively caused by the targeted mutations in all of the nine MAP kinase gene-deletion strains used.

### Vegetative morphogenetic defects in MAP kinase mutants

#### Colony phenotypes of MAP kinase gene-deletion mutants were distinct between the MAP kinase pathways, but conserved within each cascade

**Figure 4:**
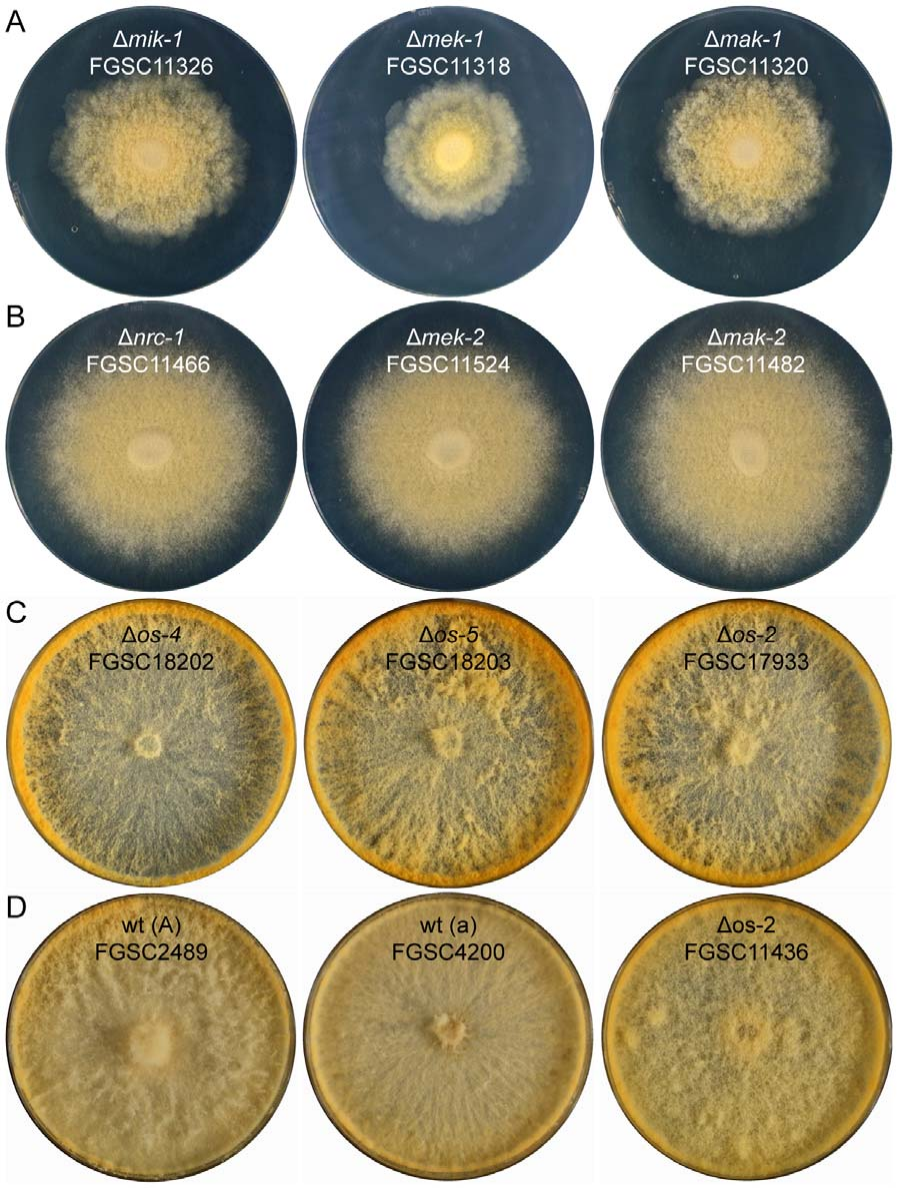
Colony morphology of MAP kinase mutants. All MAP kinase mutants showed macroscopic colony phenotypes clearly distinct from the wild type and between the three MAP kinase pathways, but highly conserved within each cascade. (**A**) CWI-MAP kinase mutants (Δ*mik-1*, Δ*mek-1* and Δ*mak-1*) typically showed increased autolysis resulting in rosette-like colony growth, and slow colony extension even on nutrient rich media. (**B**) MAP kinase mutants of the PR pathway (Δ*nrc-1*, Δ*mek-2* and Δ*mak-2*) were characterized by short aerial hyphae and conidiation starting from the colony center. (**C**) Colony phenotypes of OS-MAP kinase mutants (*Δos-4*, *Δos-5* and *Δos-2*) comprised reduced aerial hyphae in the colony center, elevated carotenoid biosynthesis and intense production of ‘sticky’ aerial hyphae and macroconidiophores were foremost at the plate edge. (**D**) Wild type controls, and the ‘old’ Δ*os-2* strain FGSC11436, which displayed a colony phenotype different to that of the genuine *os* mutants (see Figure S4 for a more detailed genotypic and phenotypic comparison between the two *Δos-2* mutants FGSC11436 and FGSC17933).

As reported earlier [40], [41], CWI-MAP kinase (Δ*mik-1*, Δ*mek-1* and Δ*mak-1*) mutants showed rosette-like colony growth caused by areas of increased mycelial autolysis as typical features (Figure 4A). Mutants of the PR-MAP kinase pathway (Δ*nrc-1*, Δ*mek-2* and Δ*mak-2*) were characterized by short aerial hyphae and conidiation starting from the colony center (Figure 4B), which was also consistent with previous findings [36], [37], [41]. MAP kinase mutants from the OS-MAP kinase pathway displayed ‘sticky’ and intensively orange-colored *macroconidiophores* (conidia-bearing hyphae) or macroconidia, with increased macroconidiation typically occurring around the edge of the culture dish rather than in the colony center (Figure 4C). The morphological alterations during conidiogenesis in *os* mutants have previously been connected to conidial lysis [41], [70], [71]. We, however, did not observe this under our tested conditions. Hyphal lysis, including ‘bleeding’ of intensely orange colored droplets, nevertheless, did occur within the vegetative mycelium (data not shown), presumably from ruptured hyphal tips [72].

### Defects during frutibody morphogenesis in MAP kinase mutants

#### Deletion of MAP kinases exclusively affected female fertility

None of the tested MAP kinase mutants generated progeny when used as a female in heterozygous crosses with a wild-type male. Whereas, ascospores were produced in reciprocal and confrontation crosses (where the mutant effectively acted as male) suggesting that male fertility, i.e. the ability of cells of a particular strain to participate successfully in a non-self fusion event leading to fertilization of an opposite mating-type female, is uncoupled from the self-fusion defect.

Where available, both mating types of a particular gene-deletion strain were tested; however, mating-type dependent effects were never evident. Decreased ascospore viability was generally observed in the progeny recovered from heterozygous crosses involving MAP kinase mutant strains, as previously reported [23]. Nevertheless, the production of meiotic progeny involving a mutant strain as male contributor unambiguously demonstrated successful completion of the sexual cycle despite the genetic defect. Impairment of ascospore germination (*a.k.a.* ascospore lethality) due to genetic defects of the mutant progeny is, by our definition, a problem associated with the subsequent vegetative growth phase.

#### Defects during protoperithecium morphogenesis were evident at the ultrastructural level

**Figure 5:**
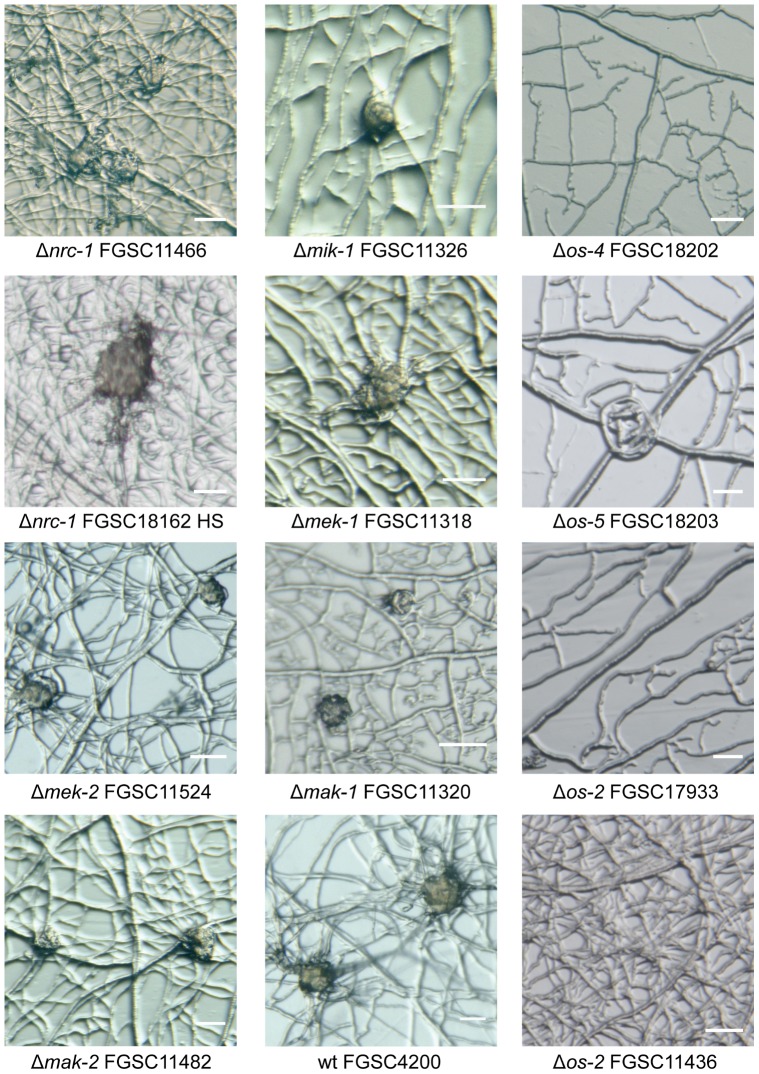
Protoperithecial development in MAP kinase gene-deletion mutants. In comparison to the wild type, which formed regular, subspherical protoperithecia 40–80 µm in diameter, only mutants of the PR- and CWI-MAP kinase cascades formed protoperithecial-like structures of similar appearance. These however, did vary in size, shape and degree of pigmentation and were not clearly discernable as protoperithecia even to an experienced microscopist using the stereomicroscopy technique shown here. It was these observations that warranted investigations using more powerful microscopic techniques, as used for Figures 1 and 6–8. Protoperithecial-like structures could not be observed in any of the newly generated OS-MAP kinase mutants. In contrast to the other *os* mutants, Δ*os-2* FGSC11436 showed disorganized mycelial architecture, typical of hyphal fusion defects. The Δ*nrc-1* strains generated from FGSC18162 by vegetative homokaryon selection (HS) showed no phenotypic differences compared to Δ*nrc-1* FGSC11466. In order to calibrate the results, all strains were inoculated onto cellophane over LSA medium (and SCM for comparison), and incubated for 5–7 days at 25°C dependent on the rate of developmental of the mutant strain. By cutting out cellophane squares carrying mycelium the same samples as shown here were subsequently prepared for LTSEM. Finally, these female cultures were fertilized with opposite mating type conidia of the wild type to confirm female sterility. All scale bars, 50 µm.

**Figure 6:**
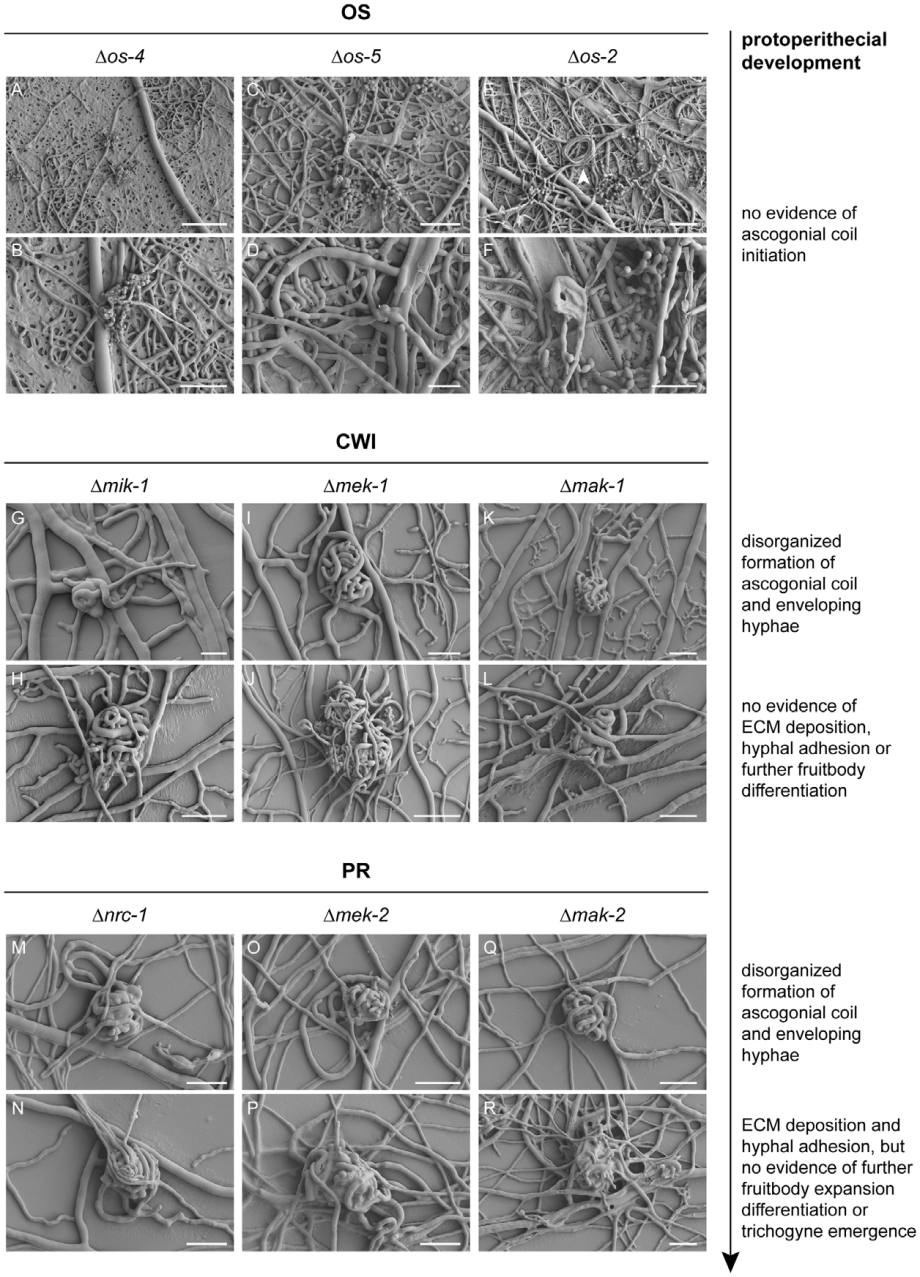
ECM and hyphal adhesion seem essential for the organized assembly of enveloping hyphae into protoperithecia. **(OS)** Despite several attempts, ascogonial coils, let alone protoperithecial-like structures, could not be identified in mycelia of the three OS-MAP kinase mutants. Large areas of the mycelium were collapsed, indicating extensive lysis of vegetative hyphae. Hyphal loops (*a.k.a.* hyphal coils or lassoes), as shown here in Δ*os-2* (arrowhead in E) were occasionally observed in all three mutants. These structures are frequently found in the wild type, and although their function is unknown, a connection to sexual development seems unlikely (see discussion). Scale bars: (A) 100 µm; (B, C, E) 50 µm; (D, F) 25 µm. **(CWI)** Δ*mik-1*, Δ*mek-1* and Δ*mak-1* strains initiated ascogonial coils and differentiated enveloping hyphae. The assembled multicellular structures, however, remained loose hyphal aggregations and ECM was absent, suggesting that hyphal adhesion was not sufficient to form subspherical protoperithecia. Scale bars: (G) 10 µm; (H, I, K, L) 25 µm; (J) 50 µm. **(PR)** Δ*nrc-1*, Δ*mek-2* and Δ*mak-2* strains produced ECM, and hyphal aggregations resembled better-organized and more spherical ‘early-stage’ protoperithecia. Nevertheless, trichogynes have not been observed in these strains, and sexual development did not progress beyond this stage. Scale bars: (M–R) 25 µm.

Assessment of female-autonomous fruitbody development by stereomicroscopy showed that six out of nine MAP kinase mutant-strains developed protoperithecium-like structures beyond the stage of ascogonial coils, the exceptions being the *os* mutants that did not initiate sexual development under the test conditions (Figure 5). In comparison to the wild type, which after 3–4 days post-inoculation formed abundant, quite regular and subspherical protoperithecia that were 80–100 µm in diameter, the frequency, size and shape of developing, protoperithecial-like hyphal aggregates varied considerably between different mutants grown under identical conditions. Fertilization of the female mutant mycelium with opposite mating-type wild-type conidia did not trigger further fruitbody differentiation. More conclusive morphological details of these structures, however, were not discernible with this simple technique and led to the employment of LTSEM for more detailed microscopic analysis. Structural definition of the developed protoperithecial precursors was generally improved when strains were cultured on LSA compared to SCM. Furthermore, the more efficient suppression of conidiogenesis on LSA in comparison to SCM greatly facilitated observations and sample preparation. Special care was taken to ensure that any unusual morphological features or altered surface properties were not due to preparation artifacts, by having an identically prepared wild-type control on each cryospecimen carrier alongside the actual samples. The sublimation of surface ice during partial freeze-drying after cryofixation [73], [74] was the key advantage over light microscopy techniques that allowed the true three-dimensional surface topology of the developing protoperithecium to be resolved (Figure 6). Key morphogenetic features typical for each of the mutants of the three MAP kinase cascades are described in the following sections.

#### OS-MAP kinase pathway: excessive ECM secretion might prevent ascogonial coil formation

The OS-MAP kinase pathway controls multiple cellular stress responses and, in sequential interaction with the CWI-MAP kinase pathway, is required for cell survival upon cell wall damage [75], [76]. Commonly reported phenotypic defects in mutants of the three central OS-MAP kinase components in filamentous fungi include: the inability to grow on hyper-osmotic medium (e.g. >3% NaCl or 1 M sorbitol); hyphal lysis; increased pigmentation of macroconidia; female sterility due to the lack of protoperithecia; and increased resistance to phenylpyrrole fungicides, such as fludioxonil or fenpliconil [70], [77], [78].

**Figure 7:**
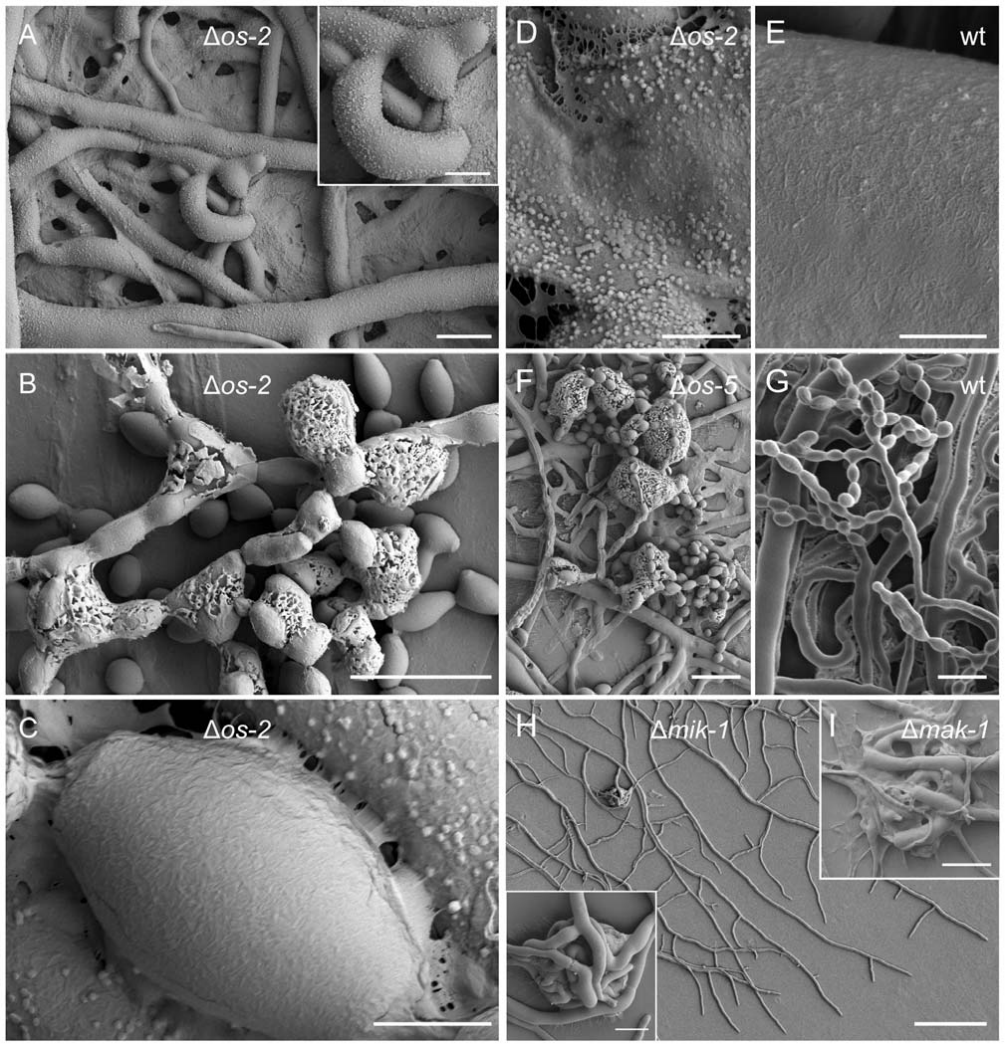
Excessive ECM deposition in OS-MAP kinase mutants and aborted fruitbody development in CWI-MAP kinase mutants. (**A**) All hyphal surfaces of Δ*os-2* (FGSC17933) were covered with punctate clusters of ECM depositions. Scale bar, 10 µm. Magnified view in inset; scale bar, 5 µm. (**B**) Macroconidiophores of Δ*os-2* were also heavily covered in ECM material. Scale bars, 20 µm. (**C**) Granular ECM depositions were not present on the surfaces of matured, detached macroconidia. Scale bar, 2 µm. (**D**) Higher magnification of the clustered ECM depositions on a mature hyphal surface of Δ*os-2*. Scale bar, 2 µm. (**E**) Smooth surface of a mature hypha of the wild type control. Scale bar, 2 µm. (**F**) ECM-covered macroconidiophore of Δ*os-5*. Scale bar, 20 µm. (**G**) Wild type macroconidiophore. Scale bar, 20 µm. (**H**) CWI-MAP kinase mutant strains displayed early-onset initiation of fruitbody development at the colony periphery. Inset shows a magnified view of the protoperithecial-like ‘hyphal knot’ formed only about 400 µm behind the leading colony edge of Δ*mik-1*. Scale bar, 100 µm; in inset 10 µm. (**I**) Immature multicellular structures in the sub-periphery of Δ*mak-1* colonies aborted, then autolyzed and were subsequently reabsorbed into the mycelium, resulting in little evidence of any recognizable protoperithecial-like structures. Scale bar, 50 µm.

Although the *N. crassa* Δ*os-4*, Δ*os-5* and Δ*os-2* gene-deletion strains analyzed in this study showed most of the above mentioned defects, our analysis could not confirm the previously reported hyphal fusion defect [41] (Figure S3). We therefore sought other reasons for the absence of protoperithecia. Despite several attempts and testing various culture conditions, we were unable to find evidence of ascogonial coils in any of the three *os* mutants (Figures 6A–6F). An interesting observation made during the SEM studies was of extensive clusters of ECM depositions covering hyphal surfaces (Figures 7A and D) and macroconidiophores (Figures 7B and 7F) of the three *os* mutants, in a way not observed in the wild-type control samples (Figures 7E and 7G). Interestingly, detached conidia, however, were free of these surface depositions (Figure 7C).

#### CWI-MAP kinase pathway: defects in hyphal aggregation and adhesion aborted protoperithecial maturation

The CWI-MAP kinase pathway senses and responds to cell-wall stress during vegetative growth, and in response to a variety of other signals including pheromone-induced morphogenesis. Common phenotypes in *mik-1*, *mek-1* and *mak-1* mutants include altered cell walls, defects in cell–cell adhesion and increased autolysis [40], [41], [79].

Our analysis showed that CWI-MAP kinase mutants clearly progressed beyond ascogonial coil formation and expansion, but failed to form tightly packed protoperithecia through organized aggregation and adhesion of enveloping hyphae (Figures 6G–6L). Protoperithecial development terminated at a loosely coiled stage that aborted, and the hyphal aggregates formed were reabsorbed into the colony, presumably fostered by increased autolysis in these mutants (Figure 7I).

Another interesting observation was early-onset of fruitbody development at the leading edge of Δ*mik-1*, Δ*mek-1*, and Δ*mak-1* colonies in plates that had been centrally inoculated (Figure 7H). This contrasted with the *N. crassa* wild type that only underwent fruitbody formation once the centrally inoculated colony had reached the edge of the culture plate. The latter occurred on SCM or LSA at 25°C in plates up to a diameter of 30 cm.

#### PR-MAP kinase pathway: activation of the PR-MAP kinase cascade occurred at the end stages of protoperithecial morphogenesis

During yeast mating, the PR-MAP kinase pathway regulates chemotropic interaction of mating partners leading to non-self fusion and fertilization (reviewed in [80]). Mutants of the orthologous PR-MAP kinases NRC-1, MEK-2 and MAK-2 of *Neurospora* have been reported to progress further in fruitbody development than mutants of the other two MAP kinase cascades, but still remained female sterile [41]. This was confirmed by our ultrastructural analysis, showing that Δ*nrc-1*, Δ*mek-2* and Δ*mak-2* strains of *N. crassa* formed densely packed protoperithecia with evidence of hyphal adhesion and normal ECM deposition on the outside (Figure 6M–6R). These mutants, however, did not show signs of trichogyne differentiation, which would be required for subsequent non-self fusion with the male mating partner.

### Localization of MAP kinases during protoperithecial development

#### Genetic complementation rescued sexual development in all three MAP kinase mutants

**Figure 8:**
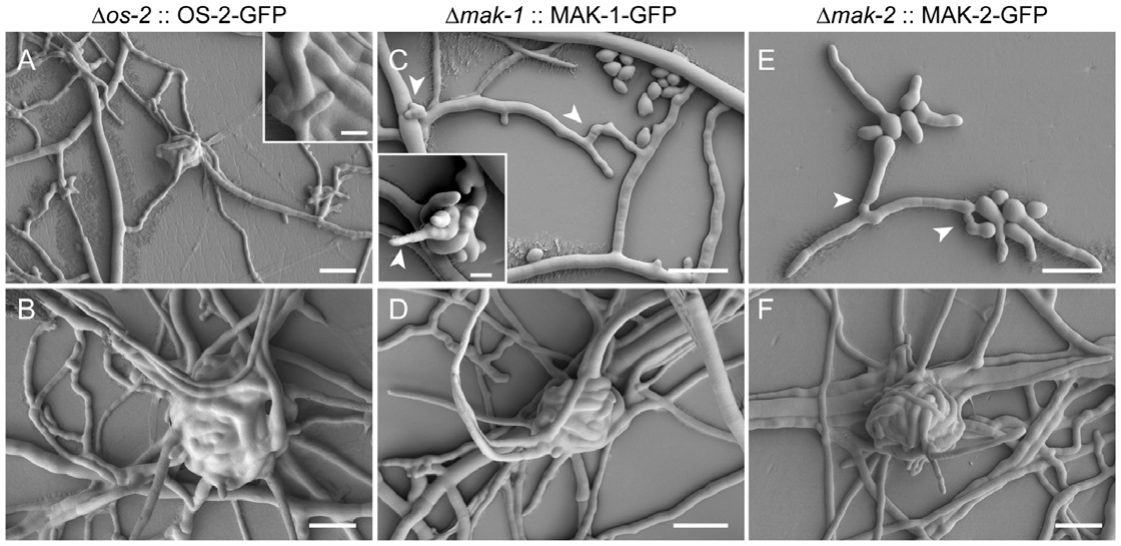
Genetic complementation rescued protoperithecial development in all three MAP kinase mutants. (**A**) Young protoperithecium of a rescued Δ*os-2* transformant (NCAL020) enwrapped by enveloping hyphae. Granulated ECM depositions as seen on Δ*os-2* hyphae (Figure 7A) could no longer be observed in the rescued Δ*os-2* transformants, which showed smooth hyphal surfaces evenly covered in ECM (compare to wild type in Figure 7E). Scale bar, 20 µm; in inset 5 µm. (**B**) Mature protoperithecium of the rescued Δ*os-2* transformant. Scale bar, 20 µm. (**C**) VHF (arrowheads) undergone in the rescued Δ*mak-1* transformant (NCAL010). Scale bar, 20 µm. The inset shows an ascogonial coil of this strain from which a straight hypha emerges which resembles a trichogyne initial (arrowhead). Scale bar, 5 µm. (**D**) Mature protoperithecium of the rescued Δ*mak-1* transformant. Scale bar, 20 µm. (**E**) CAT-mediated cell fusion (arrowheads) in a rescued Δ*mak-2* transformant (NCAL043). Scale bar, 20 µm. (**F**) Mature protoperithecium of a rescued Δ*mak-2* transformant. Scale bar, 20 µm.

Wild-type morphology was restored by ectopic expression of OS-2-GFP, MAK-1-GFP and MAK-2-GFP in Δ*os-2* (FGSC17933), Δ*mak-1* (FGSC11320) and Δ*mak-2* (FGSC11482), respectively (Figure 8). In the rescued Δ*os-2* transformants the wild-type phenotypes that were recovered included: the formation of ascogonial coils that developed into mature protoperithecia; absence of excessive ECM secretion and hyphal lysis; and turgid hyphae with smooth surfaces (Figures 8A and 8B). The vegetative hyphal fusion defects of Δ*mak-1* [41] and Δ*mak-2* strains [36], [37], [41] were also fully recovered in their respective transformants (Figures 8C and 8E), as was their ability to complete protoperithecial development (Figures 8D and 8F). When used as females in heterozygous crosses with the wild type, the rescued transformants of all three MAP kinase mutants successfully completed sexual development, producing viable ascospores. Notably, genetic complementation of Δ*os-2* FGSC11436 restored osmoresistance and fenpiclonil sensitivity, but not the hyphal fusion defect of this strain suggesting the hyphal fusion defect is uncoupled from the *os-2* deletion (Figure S4).

#### Optical sectioning of protoperithecia

**Figure 9:**
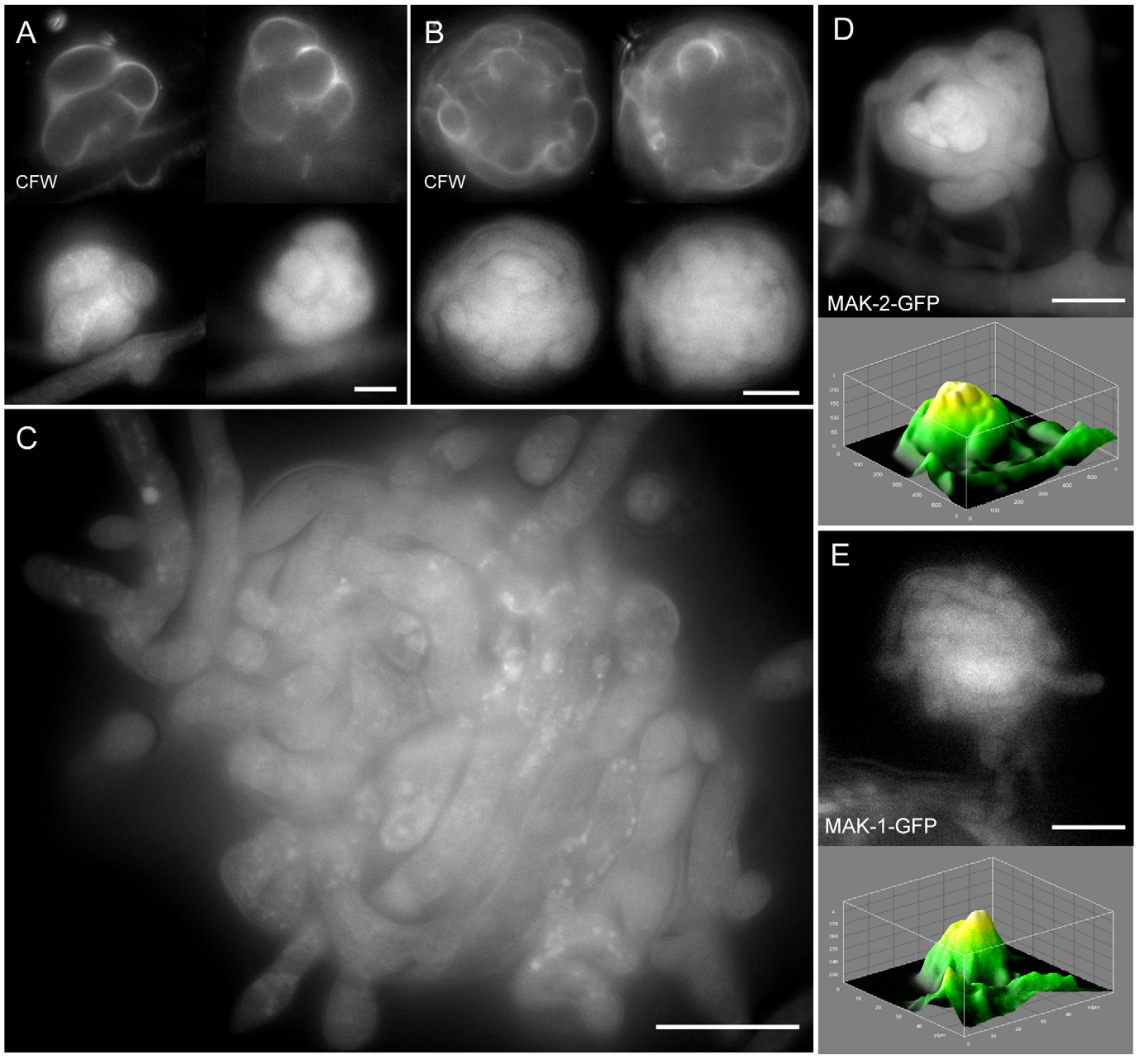
Optical sectioning of developing protoperithecia. Montages of selected optical sections through developing protoperithecia of the rescued Δ*mak-2* strain expressing MAK-2-GFP (NCAL043). (**A**) The small dimensions of a late stage ascogonial coil are fully accessible to optical sectioning when labelled with CFW and MAK-2-GFP. Scale bar, 5 µm. (**B**) With increasing size, CFW dye is unable to penetrate the interior of the developing fruitbody, and consequently cannot be used to optically section the interior of the ascogonium. Fluorescently labelled MAK-2, however, allows visualization of the whole protoperithecium. Scale bar, 10 µm. (**C**) Optical sectioning of a mature protoperithecium reveals the complex and tightly wound hyphal network comprising this structure. Scale bar, 20 µm. (**D**) Middle section of a protoperithecium expressing MAK-2-GFP. The corresponding surface plot shows that fluorescence intensity peaks in the central core region, suggesting that MAK-2-GFP accumulates in the ascogonial coil tissue. (**E**) MAK-1-GFP fluorescence in the rescued *Δmak-1* strain (NCAL010) also peaked in the central ascogonium region, however, was not as pronounced as in the case of MAK-2. Scale bar, 10 µm. Movies S5, S6, S7, and S8 show full z-stacks of optically sectioned protoperithecia.

Optical sectioning of fluorescently labelled protoperithecia revealed the tightly wound hyphal network of living fruitbodies (Figure 9). CFW staining of cell walls and expression of fluorescent fusion proteins within the cytoplasm facilitated optical sectioning of complete ascogonial coils and early-stage protoperithecia (Figure 9A). Due to the limited depth which CFW penetrates into the centre of protoperithecia, only the fluorescent fusion protein signal was able to provide detail of the internal organization of larger protoperithecia (Figure 9B). Unfortunately, this approach still did not unequivocally elucidate whether or not cell fusion had occurred between hyphae within developing protoperithecia (Figure 9C).

Interestingly, for MAK-2-GFP expressing-strains higher fluorescence could be detected within the central ascogonial-coil region, whereas signals were on average 25% lower in the adjoining enveloping hyphae and dropped another 20% in neighboring vegetative hyphae (Figure 9D). A similar trend was observed for MAK-1-GFP distribution, although with overall weaker fluorescence intensities (Figure 9E). Although OS-2-GFP could not be detected in the rescued mutant fruitbodies, it could be detected in conidial germlings (A. Lichius, unpublished data).

## Discussion

### Protoperithecial morphogenesis in the *N. crassa* wild type

To provide a baseline comparator for the analyses of MAP kinase gene-deletion mutants we commenced with a detailed description of protoperithecial morphogenesis in the wild type using live-cell imaging and LTSEM. We conclude that this process can be conveniently divided into four main morphogenetic stages of hyphal differentiation, leading from the vegetative mycelium to the fertilizable protoperithecium. Notable key aspects highlighted by our analyses include: (1) the initial stage being the ascogonial coil, a tight-helical branch with outer dimensions not exceeding 15 µm in diameter, that forms the ascogonium in the center of the developing protoperithecium; (2) septation of the ascogonial coil is a precursor to the emergence of enveloping hyphae; (3) enveloping hyphae, which differentiate as branches of either the ascogonium or one of its neighboring compartments, enlarge the structure; (4) additional enwrapping by enveloping hyphae that may originate from the surrounding vegetative mycelium determine shape, and (5) regulated deposition of extracellular matrix involved in the tight adhesion of ascogonial coil and enveloping hyphae seals the subspherical encasing of the developing fruitbody.

We also take this opportunity to clarify that ascogonial coils in *N. crassa* and related ascomycetes are developmentally different to vegetative hyphal coils (*a.k.a.* hyphal lassoes). The formation of hyphal lassoes has been described by several authors, and interpreted to be abortive ascogonial coils or ‘pseudo-ascogonia’ in *N. crassa* [81], [82], *N. tetrasperma* [83] and *S. macrospora* [84]. However, we are not aware of any evidence to support this interpretation in *N. crassa* as has been recently discussed for hyphal lassoes in *Aspergillus nidulans* [85].

The formation of a subspherical fruitbody from tubular hyphae can most efficiently be achieved by the combination of a tight coil and enwrapping branches. Hyphal lassoes are too loosely wound to generate the compact ascogonial coil observed in the center of developing protoperithecia.

### Gene deletions that impact on sexual progression mainly affected the female partner

All gene-deletion mutants investigated in this project were blocked in sexual development only when used as females in heterozygous crosses with the wild type. Using conidia of mutant strains as the male fertilizing agent for opposite mating-type, wild-type females did not block sexual development and resulted in the successful production of ascospores. Exclusive female sterility is consistent with earlier findings [36], [40], [86] and confirms asymmetry of female and male function during mating in *N. crassa* [20]. Mutant strains that are highly male-fertile, but completely female-sterile, are not surprising, as the female function is more complex and thus presents a larger mutational target than the male function. Female function is therefore more easily lost through targeted gene-disruption than is male function [87]. Thus far, the only reported example of a *N. crassa* mutant that has been shown to be female *and* male sterile is the Δ*prm-1* strain [88].

### MAP kinase mutants were defective at different stages of protoperithecial morphogenesis

Light microscopy illustrated that six out of nine mutants in this investigation formed protoperithecium-like structures that did not mature when judged by size and degree of pigmentation compared with the wild type protoperithecium. For CWI-MAP kinase mutant strains this is the first report that protoperithecium-like structures develop to these advanced stages. Furthermore, morphological differences between protoperithecium-like structures observed in CWI-MAP kinase and PR-MAP kinase mutants indicated that the deleted genes have different functions during protoperithecial morphogenesis.

Two essential characteristics of developmentally arrested mutant protoperithecia became evident: (1) enveloping hyphae, which were only arranged as loose knots with significant gaps between them, and (2) excess or absence of ECM deposition. Loose-knit protoperithecia were observed for the MAP kinase mutants of the CWI pathway. Furthermore, these mutants did not appear to deposit ECM, which markedly contrasted with MAP kinase mutants of the PR pathway and the wild type. MAP kinase mutants of the OS pathway produced excess ECM, which formed granulated clusters on all hyphal surfaces, accumulating in greater quantities on conidiophores. These latter MAP kinase mutants were characterized by the complete absence of developing fruitbodies, consistent with previous reports [89], [90].

### Controlled extracellular matrix deposition was crucial for adhesion of enveloping hyphae

Our results suggested that the organized assembly and adhesion of enveloping hyphae is essential for protoperithecium morphogenesis. If hyphal adhesion was possible to a small degree, protoperithecial development progressed further, compared to mutants where ECM deposition seemed to be deregulated. The inability of MAP kinase mutants of the OS pathway to initiate sexual development is potentially due to excessive ECM secretion on the hyphal surface, which could prevent efficient coiling and adhesion, and correlated with numerous collapsed and ‘bleeding’ hyphae, and ‘sticky’ conidiophores. MAP kinase mutants of the CWI pathway only formed loose hyphal aggregates that lacked ECM. These aggregates aborted and became quickly autolyzed PR-MAP kinase mutants progressed furthest by forming compact, organized protoperithecial-like structures. However, emergence of trichogyne-like hyphae was not observed in PR-MAP kinase mutants, suggesting that fruitbody development stalled before a mature stage was reached even in these strains. These MAP kinase pathway-dependent differences in superficial ECM deposition led us to propose that regulated ECM deposition should be regarded as a defining stage of protoperithecial development in *N. crassa*. In addition, some of the ECM produced may play an important role for the formation of conglutinate cells and pseudoparenchymatous tissues [2], [4], [5].

### Apical dominance might restrict sexual development and senescence in the colony sub-periphery

Another aspect that influenced fruitbody development and its analysis, respectively, was the early-onset of autolysis in the mycelia of CWI-MAP kinase mutants. This phenomenon has been reported for a number of homologous strains in different fungal species and is generally regarded as one determining feature of fungal cell-wall mutants [41], [79], [91]. Autolysis of immature protoperithecia occurred in the wild type, but much less frequently. Early autolysis resulted in very quick degradation of aborted protoperithecia in the center and sub-periphery of colonies of all CWI-MAP kinase mutants of *N. crassa*. As a likely consequence of this, intact protoperithecial aggregates could only be located and analyzed close to the colony edge, interestingly, even before the mycelium had reached the edge of the culture dish. In the wild type or other developmental mutants we studied, it was not common to observe protoperithecial formation commencing before tips of peripheral leading-hyphae had encountered the edge of the Petri dish. Premature entry into sexual development and early-onset autolysis of the initiated fruitbodies are both signs of accelerated senescence of these mutants. Wild type colonies can extend to considerable sizes before initiating protoperithecial development in response to physical confinement at the colony edge. This ‘edge-effect’ stimulus of fruitbody formation has been documented for species closely-related to *N. crassa* [92], including *S. macrospora* [84], [93] and *S. brevicollis* [94], supporting the hypothesis that apical dominance of the leading-edge of the actively growing colony suppresses ascogonial coil initiation, and consequently delays aging in the sub-periphery of the wild type. The particular phenotype of increased senescence in CWI-MAP kinase mutants greatly impeded experimental analysis of fruitbody development, but provided one possible explanation why protoperithecial initials of these mutants had escaped detection by earlier investigators.

### Trichogyne emergence concludes protoperithecial development

MAP kinase mutants of the PR pathway (*nrc-1*, *mek-2* and *mak-2*) have previously been reported to progress further in fruitbody development than mutants of the other two MAP kinase cascades [41]. This notion was confirmed by our analysis, which due to the absence of trichogyne-like hyphae, further suggests that the PR pathway might also act during the transition from protoperithecial to perithecial development. Thus, signaling through the PR pathway may not exclusively be involved in trichogyne homing and mating-cell fusion, but also in trichogyne initiation. As trichogynes are formed in the wild type in the absence of opposite mating-type pheromone, the involvement of a self-signaling molecule, which triggers morphogenetic transitions up until this point, is indicated. Crosstalk between all three *Neurospora* MAP kinase pathways during regulation of female sexual development is very likely as suggested previously [40], [41].

Taken together, we propose that deregulated MAP kinase signaling leads to the inability to develop protoperithecia in an organized manner, i.e. to assemble a tightly wound ascogonial coil with enveloping hyphae ‘hugging’ its surface through adhesion and regulated ECM deposition. Hyphal attachment following tip-growth arrest is a precursor to vegetative hyphal fusion (VHF) and might provide an important trigger for the activation of the fusion machinery [95]. The lack of contact-induced tip growth arrest and hyphal tip attachment is a commonly observed phenotype in VHF mutants [96], including CWI- and PR-MAP kinase gene-deletion strains. Therefore, the inability to attach and trigger the transition to the next morphogenetic stage may provide a functional connection between VHF and fruitbody development. In the event that certain developmental checkpoints are not reached, further fruitbody morphogenesis is aborted and the material becomes reabsorbed and recycled within the colony. This has important implications for the preparation and timing of experimental analysis of protoperithecial-defective strains.

### The role of self-fusion during protoperithecial morphogenesis remains unclear

All nine MAP kinase mutants analyzed in this study were defective in sexual development, but only six were defective in vegetative hyphal self-fusion. The link between vegetative cell fusion and sexual development has repeatedly been made (e.g. [23], [67], [69]) but whether self-fusion events are involved in the formation of the fully functioning/co-operative multicellular structure of the protoperithecium has not been established. Unfortunately, despite very careful observations, we were unable to clearly identify self-fusion connections within the developing protoperithecium. An obstacle for these analyses is that unless one can actually observe the fusion-process occurring, it is difficult to reliably differentiate a fusion event from a septation event inside the fruitbody. Our findings from optically sectioning early-stage protoperithecia suggest that MAK-2 and MAK-1 participate in differentiation processes inside the ascogenous tissue. Considering the importance and presence of both MAP kinases at germling fusion sites in *N. crassa* [96], [97], this leaves the possibility for cell fusion to occur in the developing protoperithecium. OS-2, on the other hand, was not detected in ascogenous tissue, nor was it detected at advanced stages of fruitbody differentiation following ascogonial coil initiation. Fusion between dissected-out *paraphyses* (sterile hyphae that grow between asci) in perithecia has been observed in *S. macrospora* [2] and *N. crassa* (K.M. Lord & N.D. Read, unpublished results), and in ascogenous tissue cell-fusion occurs in croziers [22]. Combined with results of independent studies that recently identified additional hyphal fusion mutants of *N. crassa* with normal protoperithecial development [69], [98], we can conclude that: (1) not all genes essential for VHF are required for protoperithecial development, and thus signaling processes regulating fusion in both processes are likely to be different, and (2) consequently, proteins that are essential in both processes might regulate different cellular events, not necessarily connected to cell fusion.

## Conclusions

Our analysis indicates that MAP kinase gene deletions did not lead to the disruption of protoperithecial development at a conserved stage, but that blockage occurred at distinct stages dependent on the affected MAP kinase cascade. The loss of an individual MAP kinase could not be compensated within the MAP kinase signalling network, further supporting the notion that each cascade functions during a specific stage of protoperithecium development and in a sequential manner. The morphogenetic phenotypes of the nine MAP kinase mutants in *Neurospora* suggests that the OS and CWI pathways act upstream of the PR pathway. The successful phenotypic rescue of all three terminal MAP kinase mutants proved that signaling through all three cascades is essential for perithecial development. The finding that *os* genes are dispensable for hyphal fusion, while essential for protoperithecial morphogenesis, complicates our attempts to understand whether hyphal fusion is required for fruitbody formation. However, this does not exclude the possibility that hyphal fusion is required, because fusion events inside the developing protoperithecium are likely to be regulated differently than VHF in the mature mycelium.

Finally, this study highlights that MAP kinases play roles in some of the key processes involved in the early stages of multicellular development in fungi, particularly: extracellular matrix deposition; hyphal adhesion; and hyphal envelopment, during the construction of protoperithecia. These are fundamental features displayed by fungi achieving the multicellular state by hyphal aggregation. The evolution of fungi with multicellular differentiated tissues has been estimated to have occurred at least 500 million years ago, to have occurred independently in the Ascomycota and Basidiomycota [99], and possibly more than once in the Ascomycota [100]. Indeed, the morphology of the perithecium has been shown from fossil evidence to be conserved for over 400 million years [101], [102]. In the future, it will be extremely important to determine the molecular basis of how fungi achieve multicellularity, in order that we can identify and analyse the key molecules (e.g. cell adhesion molecules) involved in this process. It will also be interesting to determine whether fungi share some of this molecular machinery with animals, from which they have been estimated to diverge 2,635 million years ago [103]. The analysis of multicellular development in experimentally and genetically tractable fungi such as *N. crassa* and *S. macrospora* should provide useful models from which to gain significant insights into these processes in even more complex eukaryotes [2].

## Supporting Information

Figure S1 **PCR-genotyping set up 1.** (**A**) Schematic overview of the PCR set up initially used for genotypic verification of gene-deletion strains. (**a**) Simplified representation of the gene-deletion process by homologous recombination. The open reading frame (ORF) of the *hph* cassette and the target gene ‘X’ are in antisense. As macroconidia, which were used for KO cassette transformation, are multinucleate, gene deletion does not necessarily occur in all nuclei of a single spore. This may result in heterokaryotic cells that contain both wild type and deletion mutant nuclei, therefore being hygromycin B resistant, but still expressing a wild type copy of the target gene X. (**b**) Presence of the target gene X in the genome of an individual strain was analyzed using one gene-specific primer positioned inside the wild type ORF (X_300_fw) paired up with a primer positioned inside the 3′ flank used for homologous recombination (3r_X). In case, PCR 1 produces a fragment of the predicted size, the target gene is still present in that strain. The 3r_x primer was used together with a matching forward primer (3f_X) to amplify a fragment of the 3′-flank as internal PCR control (PCR 2). (**c**) Presence of the *hph*-cassette in the target locus was analyzed with PCR 3, using the *hph*-cassette-specific hph_300_fw primer paired up with 3r_X. Again as internal PCR control (PCR 4) hph_300_rv was used together with hph_test_fw to amplify a part of the *hph* gene. Due to the poor binding capacities of hph_test_fw, which consequently gave only very little product (all PCR 4 bands are rather weak), this approach was soon abandoned and replaced by the improved PCR genotyping set up 2 shown in Figure S2. Green arrows represent forward primers, red arrows represent reverse primers. The *hph*-cassette is oriented in antisense relative to the target gene locus. Therefore, in PCRs 3 two seemingly non-matching reverse primers were used. (**B**) Functionality of all genotyping primers was initially verified using wild type gDNA template. (**C**) According to the schematics in (A) presence of the *hph*-cassette in the targeted loci could be confirmed for all six MAP kinase gene-deletion mutants of the PR-MAP kinase and CWI-MAP kinase pathway. The *hph*-specific PCRs 4 did only result little product, if at all. However, functional presence of that gene is sufficiently backed up by the fact that all strains grew on 100 µg/ml hygromycin B. Extremely weak, residual wild type background could only be detected for Δ*nrc-1* and Δ*mak-2*, but not for any of the other four kinase mutants. This residual wild-type background, however, was in no case sufficient to rescue the dominant mutant phenotype, which was identical amongst all three PR-MAP kinase mutants (Figure 4B). Furthermore, the wild type-like phenotype of the heterokaryotic Δ*nrc-1* FGSC18162 replacement strain switched to a mutant phenotype identical to that of Δ*nrc-1* FGSC11466 after the first generation of vegetative homokaryon purification in eight of ten isolated clones providing sufficient proof for their correctness (data not shown). (**D**) All three PR-MAP kinase mutants (Δ*nrc-1* FGSC11466, Δ*mek-2* FGSC11524 and Δ*mak-2* FGSC11482) have been confirmed to still carry the Δ*mus-51* deletion, whereas, all three CWI-MAP kinase mutants (*Δmik-1* FGSC11326, Δ*mek-1* FGSC11318 and Δ*mak-1* FGSC11320) have been complemented in that gene after back-crossing. This is consistent with the genotypic annotation of these strains in the master spreadsheet maintained at the Dartmouth *Neurospora* Genomics Project site. In Δ*mek-2* FGSC11524 homokaryon purification has been accomplished not by back-crossing, but vegetatively through single spore isolation of uninuclear microconidia. Consequently, the Δ*mus-51* could not be complemented by the mating partner. Due to low viability of the progeny after back-crossing Δ*nrc-1* FGSC11466 and Δ*mak-2* FGSC11428 strains had to be deposited as heterokaryons. To date, there are no reports that deletion of *mus-51* or *mus-52* in *Neurospora*, or other *ku70*- or *ku80*-orthologous deletions in other fungi, have an influence on the phenotype, apart from their intended molecular effect of eliminating non-homologous end-joining recombination [104]. Therefore, presence of these mutations is not expected to influence phenotypic analyses of this study in any way.
(TIF)

Figure S2 **PCR-genotyping set up 2.** (**A**) Schematic overview of the improved PCR set up used for genotypic verification of gene-deletion strains. (**a**) Simplified representation of the gene-deletion process by homologous recombination. For detailed description please refer to Figure S1 legend. (**b**) Presence of the target gene X in an individual strain was analyzed using two gene-specific primers positioned inside the wild type ORF (X_1000_rv and X_500_fw) paired up with primers positioned outside of the 5′ and 3′ flank used for homologous recombination (X-5′200_fw and X-3′300_rv). If PCR 1 and PCR 2 produced amplicons of the predicted size, then a copy of the target gene was still present in that strain. (**c**) Presence of the *hph*-knock-out cassette in the target locus was analyzed with primers specific for the cassette (hph_800_fw and hph_300_rv), paired up with the same primers outside of both flanks as described before. Analogous to (b), products from PCR 3 and PCR 4 confirmed that the *hph*-cassette has been integrated exactly at the targeted location in the genome, and thus replaced the native gene. Amplification products from PCRs 1–4 have relative size differences of at least 200 bp to one another, in order to facilitate differentiation after gel-electrophoresis. Green arrows represent forward primers, red arrows represent reverse primers. The *hph*-KO cassette is oriented in antisense relative to the target gene locus. Therefore, in PCRs 3 and 4 two seemingly non-matching reverse primers were used. (**B–D**) Genotypic verification of *os* mutants. All primers were initially tested using wild type gDNA template (left column). In all ‘new’ Δ*os-2*, Δ*os-4*, and Δ*os-5* mutants the *hph*-KO cassette has replaced the targeted gene at its specific locus, confirming that the genes were correctly removed through homologous recombination. The ORFs of the targeted genes could not be detected somewhere else in the genomes of Δ*os-2* and Δ*os-4*, respectively, whereas, in Δ*os-5* weak bands in PCRs 1, 2 and the *os-5*-specific amplicon indicate residual presence of the wild type locus, suggesting that this strain is still heterokaryotic. Nevertheless, this extremely weak wild-type background was not enough to alter the dominant mutant phenotype of strain Δ*os-5* FGSC18203, which notably was 100% consistent with that of Δ*os-4* FGSC18202 and Δ*os-2* FGSC17933 (Figure 4C). The presence of *mus-52* was found in all three Δ*os* mutant strains, whereas *mus-51* was absent from Δ*os-4* and Δ*os-5* indicating that only Δ*os-2* has successfully been recovered from back-crossing. As outlined in the master spread-sheet of the *Neurospora* Genome Project, Δ*os-4* FGSC18202 and Δ*os-5* FGSC18203 have been homokaryon-purified vegetatively by isolation of hygromycin B-resistant microconidia, and thus Δ*mus-51* could not be complemented. However, apart from its intended molecular phenotype (eliminated non-homologous recombination) no further macro- or microscopic phenotypes are known to be caused by these mutations. A pair of primers amplifying a part of the actin locus was used as internal PCR controls, generating a 700 bp fragment in each reaction. Indicated PCRs 1 and 2 refer to the PCR set up shown in (A).
(TIF)

Figure S3 **Phenotypic characterization of OS-MAP kinase mutants.** A by-product of our morphogenetic analyses of protoperithecial development was the finding that vegetative hyphal fusion in the mature colony and between conidial germlings of the OS-MAP kinase mutants Δ*os-4* FGSC18202, Δ*os-5* FGSC18203 and Δ*os-2* FGSC17933 mutants was functional and indistinguishable from the wild type. (**A**) VHF in the mature colony of all three *os* mutants (Δ*os-4*, FGSC18202; Δ*os-5*, FGSC18203 and Δ*os-2*, FSGC17933) was indistinguishable from the wild type controls (mat *A*, FSGC2489 and mat *a*, FGSC4200). A fusion defect was only observed in the ‘old’ Δ*os-2* FGSC11436 strain. See Movies S1, S2, S3, and S4 for hyphal fusion phenotypes of these strains. (**B**) Fusion competency of the ‘new’ *os* mutants was furthermore confirmed in germling-fusion assays. Again, only FGSC11436 was found to be cell fusion defective. Arrowheads indicate fusion connections. All scale bars, 10 µm. As this contradicted previous reports [41], we wanted to confirm the genuine phenotype of these strains by testing two additional, well-documented characteristics of OS-MAP kinase mutants: osmosensitivity and resistance to phenylpyrrole fungicides [70]. All three ‘new’ *os* mutant strains showed increased osmosensitivity under salt stress, and increased resistance against the fungicide fenpiclonil, confirming that they are genuine OS-MAP kinase mutants of *N. crassa*. Ectopic expression of OS-2-GFP restored osmotolerance and fungicide sensitivity in Δ*os-2* transformants (NCAL020-1 and -2). (**C**) Whereas, the wild type strains were able to grow in the presence of 6% NaCl, colony extension in all three *os* mutants was significantly impaired already in the presence of 3% salt, and completely blocked with 6% NaCl in the medium. (**D**) Only the three *os* mutants were resistant to the fungicide, whereas resistance was lost in the genetically complemented Δ*os-2*::OS-2-GFP strains. (**E**) In comparison to the VMM controls, CAT-mediated cell fusion was significantly reduced in the *os* mutants in the presence of 3% NaCl, which was recovered close to wild-type levels in the rescued Δ*os-2*::OS-2-GFP transformants (NCAL020-1 and -2). Germling fusion was effectively inhibited through 1.5 µM fenpiclonil in the wild type and rescued Δ*os-2*::OS-2-GFP transformants, but unaffected in the three genuine *os* mutants. Taken together, these findings confirm that Δ*os-4*, FGSC18202; *Δos-5*, FGSC18203 and Δ*os-2*, FSGC17933 are genuine osmosensitive MAP kinase mutants, and that they are dispensable for cell fusion in *N. crassa*.
(TIF)

Figure S4 **Phenotypic and genotypic characterization of Δ** ***os-2*** **FGSC11436.** (**A**) Differences in colony development, measured as radial colony extension, under salt and fungicide stress were compared between wild type, Δ*os-2* FGSC11436, Δ*os-2* FGSC17933 and corresponding genetically complemented transformants NCAL018 and NCAL020, respectively. The wild type was able to grow in the presence of 6% w/v NaCl in the medium, but unable to grow in the presence of the fungicide fenpiclonil (1.5 and 4.5 µM). This behavior was not altered through ectopic expression of OS-2-GFP in the wild type-transformant strains NCAL016-1 and -2. Due to its hyphal fusion defect, colony development of Δ*os-2* strain FGSC11436 was slower on the control medium. In addition, this strain showed increased sensitivity to salt stress and was unable to grow on medium supplemented with 6% NaCl. However, it was resistant to fenpiclonil. Osmosensitivity was rescued through ectopic expression of OS-2-GFP in the FGSC11436 transformants NCAL018-2 and -3, and its sensitivity to fenpiclonil was restored. With respect to osmosensitivity and fenpiclonil resistance, Δ*os-2* strain FGSC17933 and its corresponding OS-2-GFP transformants NCAL020-1 and -2, respectively, showed identical characteristics. (**B**) Normal hyphal morphology at the colony periphery of Δ*os-2* FGSC11436 under non-stress conditions and in the wild type in the presence of 6% salt. In the presence of 3% salt, both Δ*os-2* mutants show swollen hyphae and irregular growth pattern. Scale bars, 100 µm. (**C**) Multiplex PCR confirming that the native *os-2* locus has been correctly exchanged for the *hph*-knock-out cassette. The 5′ os-2 signal is of the wrong size and thus most likely an unspecific product. Alternatively, this signal might indicate an unintended genetic alteration at the 5′-flank of the *os-2* locus. Indicated PCRs 1-4 refer to the PCR set up shown in Figure S2. (**D**) Genetic complementation of Δ*os-2* FGSC11436 with *os-2-gfp* did not rescue the central-conidiation phenotype, nor did it rescue the VHF defect of that strain. Collectively, the results strongly suggest that although the *os-2* gene has been correctly deleted in FGSC11436, leading to increased osmosensitivity and fenpiclonil resistance, the cell fusion defect must be caused by an additional, unintended genetic alteration in that strain, and thus is not part of the genuine *os* mutant phenotype.
(TIF)

Table S1 **Oligonucleotides used for PCR-genotyping.** Please refer to Figures S1 and S2 to deduce primer positions and pairing.
(DOCX)

Movie S1 **Time-courses of successful VHF in the ‘correct’ Δ** ***os*** **mutants is easily revealed by cytoplasmic streaming through fusion connections.** Movie S1 showing Δ*os-5* FGSC18203, Movie S2 showing Δ*os-4* FGSC18202, and Movie S3 showing Δ*os-2* FGSC17933.
(MP4)

Movie S2 **Time-courses of successful VHF in the ‘correct’ Δ** ***os*** **mutants is easily revealed by cytoplasmic streaming through fusion connections.** Movie S1 showing Δ*os-5* FGSC18203, Movie S2 showing Δ*os-4* FGSC18202, and Movie S3 showing Δ*os-2* FGSC17933.
(MP4)

Movie S3 **Time-courses of successful VHF in the ‘correct’ Δ** ***os*** **mutants is easily revealed by cytoplasmic streaming through fusion connections.** Movie S1 showing Δ*os-5* FGSC18203, Movie S2 showing Δ*os-4* FGSC18202, and Movie S3 showing Δ*os-2* FGSC17933.
(MP4)

Movie S4 **Time-course showing the fusion incompetence in the ‘wrong’ Δ** ***os-2*** **strain FGSC11436, whose fusion hyphae ignore each other, and lack the growth arrest response upon physical contact required to establish a fusion connection.**
(MP4)

Movie S5 **Z-stack sequence of an optically sectioned late-stage ascogonial coil, expressing MAK-2-GFP, stained with the cell-wall marker dye CFW.**
(MP4)

Movie S6 **Z-stack sequence of an optically sectioned early stage protoperithecium, expressing MAK-2-GFP, stained with the cell-wall marker dye CFW.**
(MP4)

Movie S7 **Z-stack sequence of an optically sectioned mature protoperithecium, expressing MAK-2-GFP.**
(MP4)

Movie S8 **Z-stack sequences of optically sectioned protoperithecium expressing MAK-2-GFP, which shows elevated accumulation in the center.**
(MP4)
