# Supplementary figures and images for: Importance of MAP Kinases during Protoperithecial Morphogenesis in Neurospora crassa
Source: PLoS One. 2012 Aug 10;7(8):e42565. doi: 10.1371/journal.pone.0042565 (PMC3416862; doi:10.1371/journal.pone.0042565)

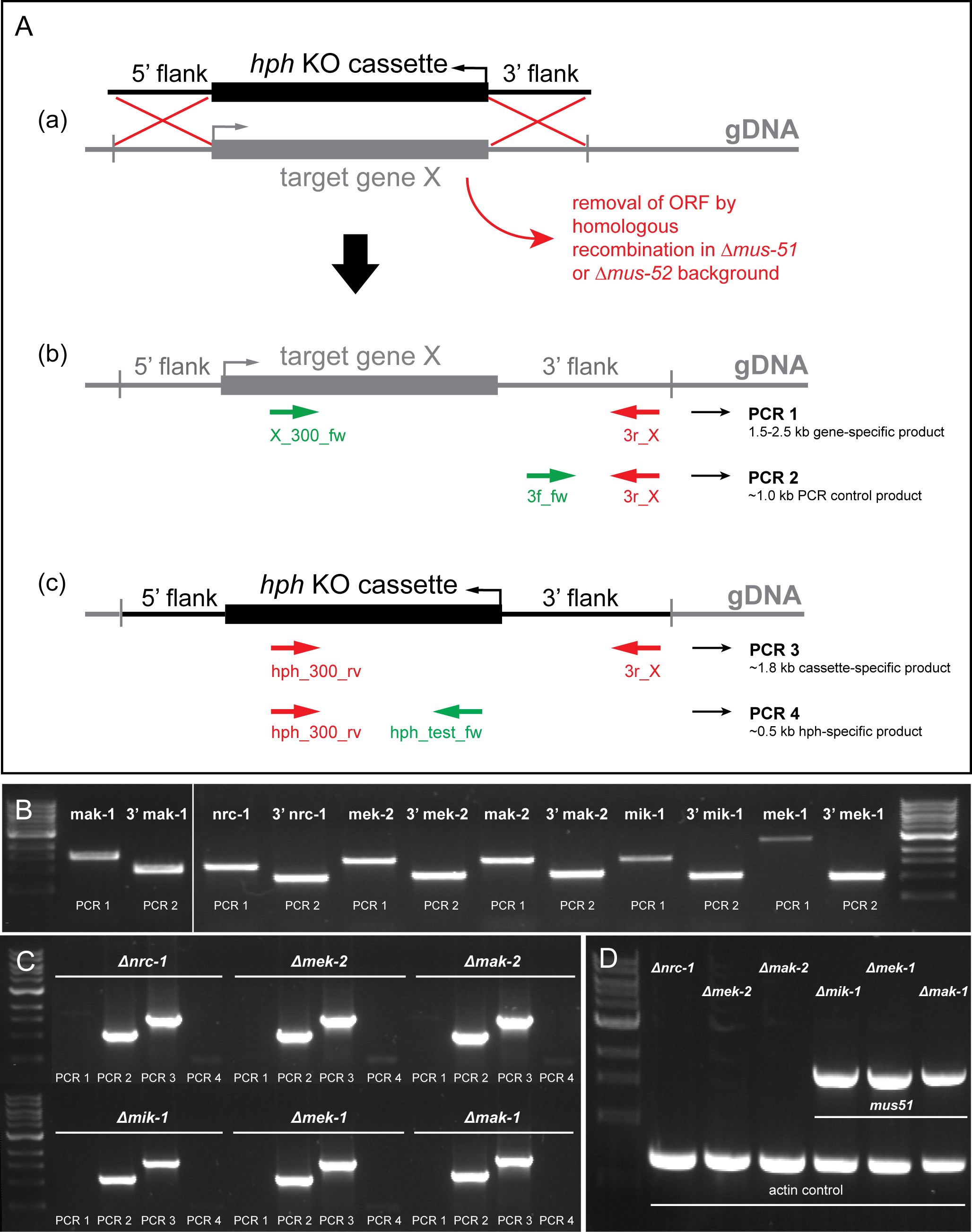

Supplement: Figure S1 — PCR-genotyping set up 1. (A) Schematic overview of the PCR set up initially used for genotypic verification of gene-deletion strains. (a) Simplified representation of the gene-deletion process by homologous recombination. The open reading frame (ORF) of the hph cassette and the target gene ‘X’ are in antisense. As macroconidia, which were used for KO cassette transformation, are multinucleate, gene deletion does not necessarily occur in all nuclei of a single spore. This may result in heterokaryotic cells that contain both wild type and deletion mutant nuclei, therefore being hygromycin B resistant, but still expressing a wild type copy of the target gene X. (b) Presence of the target gene X in the genome of an individual strain was analyzed using one gene-specific primer positioned inside the wild type ORF (X_300_fw) paired up with a primer positioned inside the 3′ flank used for homologous recombination (3r_X). In case, PCR 1 produces a fragment of the predicted size, the target gene is still present in that strain. The 3r_x primer was used together with a matching forward primer (3f_X) to amplify a fragment of the 3′-flank as internal PCR control (PCR 2). (c) Presence of the hph-cassette in the target locus was analyzed with PCR 3, using the hph-cassette-specific hph_300_fw primer paired up with 3r_X. Again as internal PCR control (PCR 4) hph_300_rv was used together with hph_test_fw to amplify a part of the hph gene. Due to the poor binding capacities of hph_test_fw, which consequently gave only very little product (all PCR 4 bands are rather weak), this approach was soon abandoned and replaced by the improved PCR genotyping set up 2 shown in Figure S2. Green arrows represent forward primers, red arrows represent reverse primers. The hph-cassette is oriented in antisense relative to the target gene locus. Therefore, in PCRs 3 two seemingly non-matching reverse primers were used. (B) Functionality of all genotyping primers was initially verified using wild type g [file pone.0042565.s001.tif]

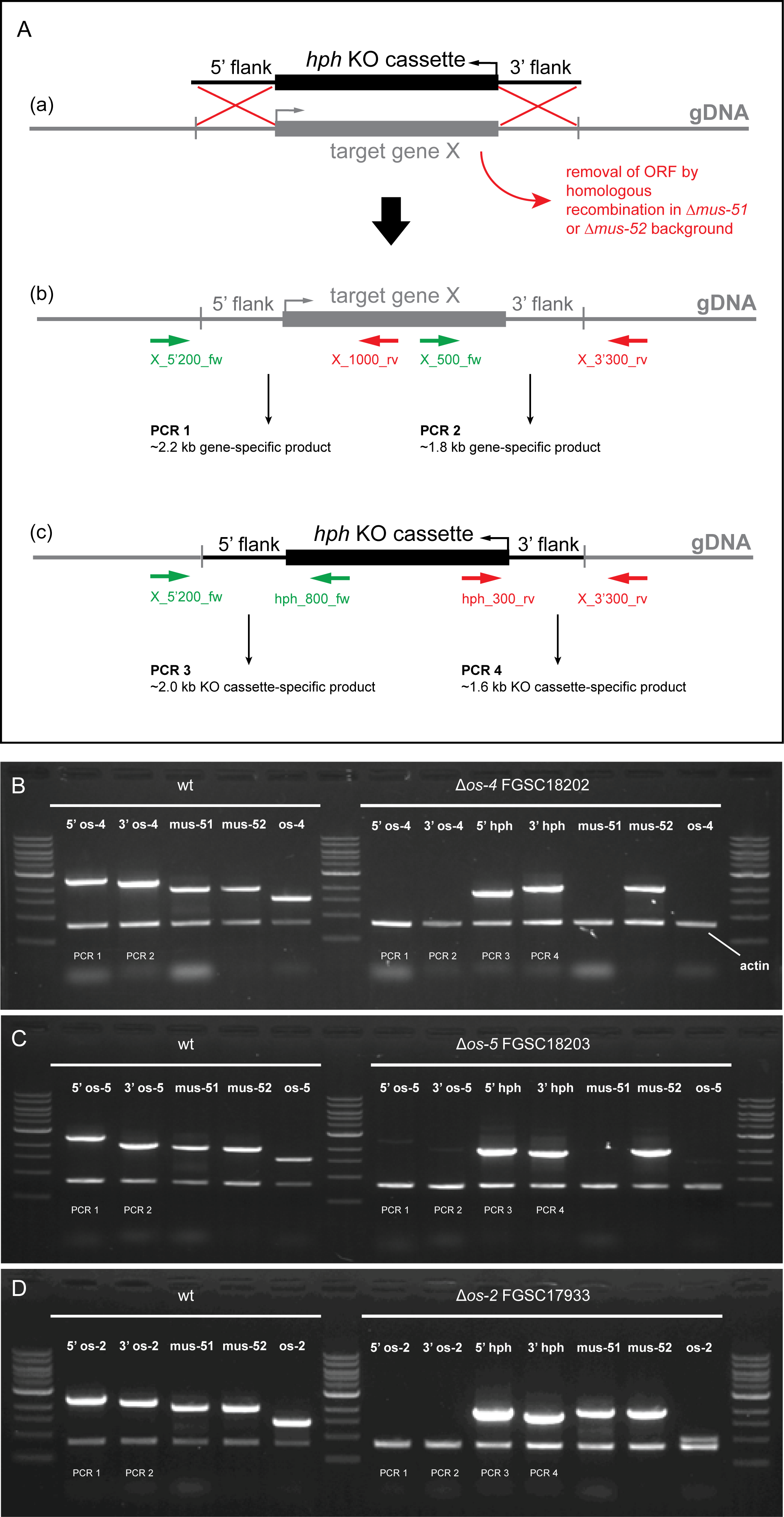

Supplement: Figure S2 — PCR-genotyping set up 2. (A) Schematic overview of the improved PCR set up used for genotypic verification of gene-deletion strains. (a) Simplified representation of the gene-deletion process by homologous recombination. For detailed description please refer to Figure S1 legend. (b) Presence of the target gene X in an individual strain was analyzed using two gene-specific primers positioned inside the wild type ORF (X_1000_rv and X_500_fw) paired up with primers positioned outside of the 5′ and 3′ flank used for homologous recombination (X-5′200_fw and X-3′300_rv). If PCR 1 and PCR 2 produced amplicons of the predicted size, then a copy of the target gene was still present in that strain. (c) Presence of the hph-knock-out cassette in the target locus was analyzed with primers specific for the cassette (hph_800_fw and hph_300_rv), paired up with the same primers outside of both flanks as described before. Analogous to (b), products from PCR 3 and PCR 4 confirmed that the hph-cassette has been integrated exactly at the targeted location in the genome, and thus replaced the native gene. Amplification products from PCRs 1–4 have relative size differences of at least 200 bp to one another, in order to facilitate differentiation after gel-electrophoresis. Green arrows represent forward primers, red arrows represent reverse primers. The hph-KO cassette is oriented in antisense relative to the target gene locus. Therefore, in PCRs 3 and 4 two seemingly non-matching reverse primers were used. (B–D) Genotypic verification of os mutants. All primers were initially tested using wild type gDNA template (left column). In all ‘new’ Δos-2, Δos-4, and Δos-5 mutants the hph-KO cassette has replaced the targeted gene at its specific locus, confirming that the genes were correctly removed through homologous recombination. The ORFs of the targeted genes could not be detected somewhere else in the genomes of Δos-2 and Δos-4, respectively, whereas, in Δos-5 weak bands in PCRs 1, 2 and the [file pone.0042565.s002.tif]

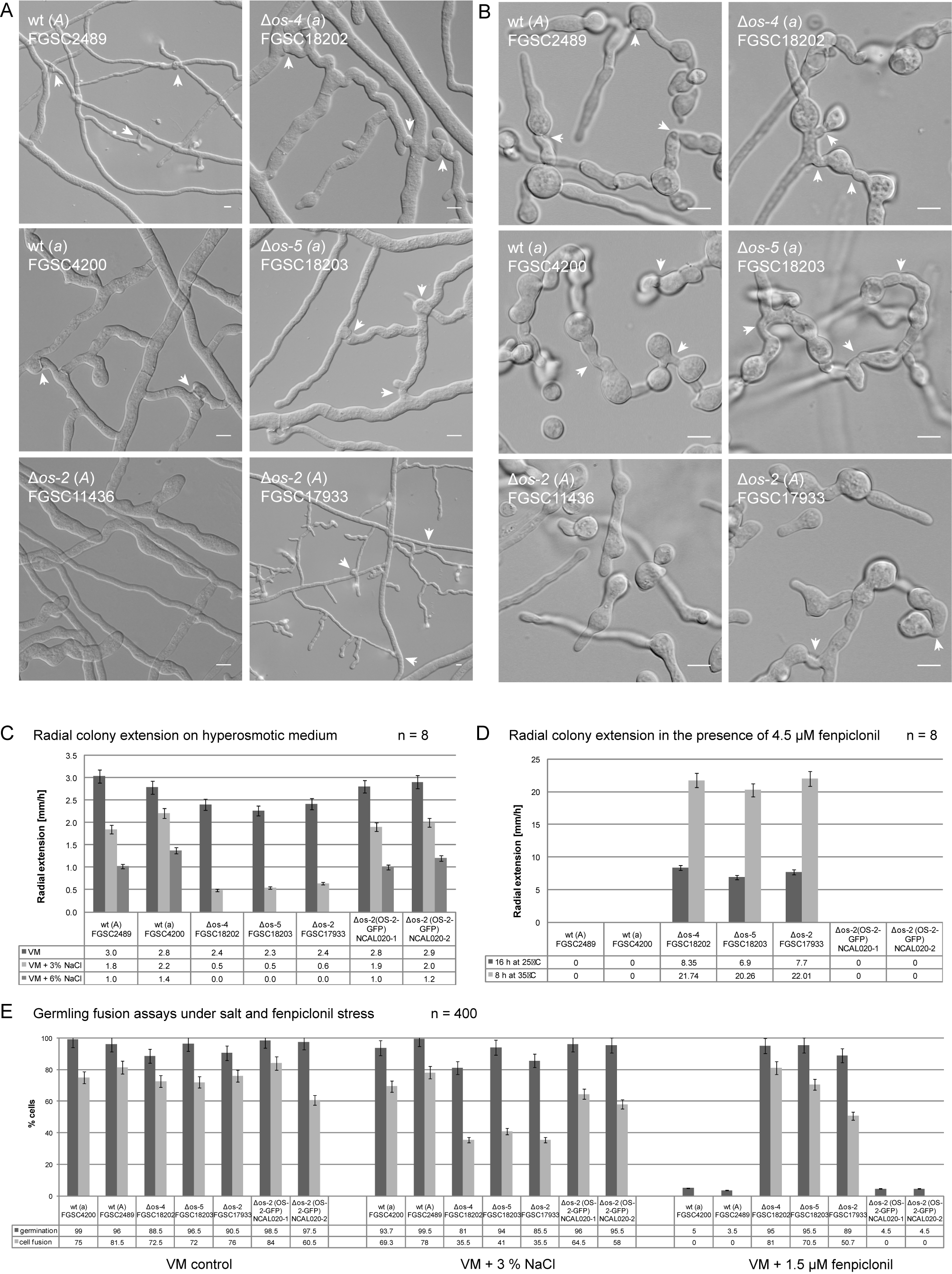

Supplement: Figure S3 — Phenotypic characterization of OS-MAP kinase mutants. A by-product of our morphogenetic analyses of protoperithecial development was the finding that vegetative hyphal fusion in the mature colony and between conidial germlings of the OS-MAP kinase mutants Δos-4 FGSC18202, Δos-5 FGSC18203 and Δos-2 FGSC17933 mutants was functional and indistinguishable from the wild type. (A) VHF in the mature colony of all three os mutants (Δos-4, FGSC18202; Δos-5, FGSC18203 and Δos-2, FSGC17933) was indistinguishable from the wild type controls (mat A, FSGC2489 and mat a, FGSC4200). A fusion defect was only observed in the ‘old’ Δos-2 FGSC11436 strain. See Movies S1, S2, S3, and S4 for hyphal fusion phenotypes of these strains. (B) Fusion competency of the ‘new’ os mutants was furthermore confirmed in germling-fusion assays. Again, only FGSC11436 was found to be cell fusion defective. Arrowheads indicate fusion connections. All scale bars, 10 µm. As this contradicted previous reports [41], we wanted to confirm the genuine phenotype of these strains by testing two additional, well-documented characteristics of OS-MAP kinase mutants: osmosensitivity and resistance to phenylpyrrole fungicides [70]. All three ‘new’ os mutant strains showed increased osmosensitivity under salt stress, and increased resistance against the fungicide fenpiclonil, confirming that they are genuine OS-MAP kinase mutants of N. crassa. Ectopic expression of OS-2-GFP restored osmotolerance and fungicide sensitivity in Δos-2 transformants (NCAL020-1 and -2). (C) Whereas, the wild type strains were able to grow in the presence of 6% NaCl, colony extension in all three os mutants was significantly impaired already in the presence of 3% salt, and completely blocked with 6% NaCl in the medium. (D) Only the three os mutants were resistant to the fungicide, whereas resistance was lost in the genetically complemented Δos-2::OS-2-GFP strains. (E) In comparison to the VMM controls, CAT-mediated cell fusion was significant [file pone.0042565.s003.tif]

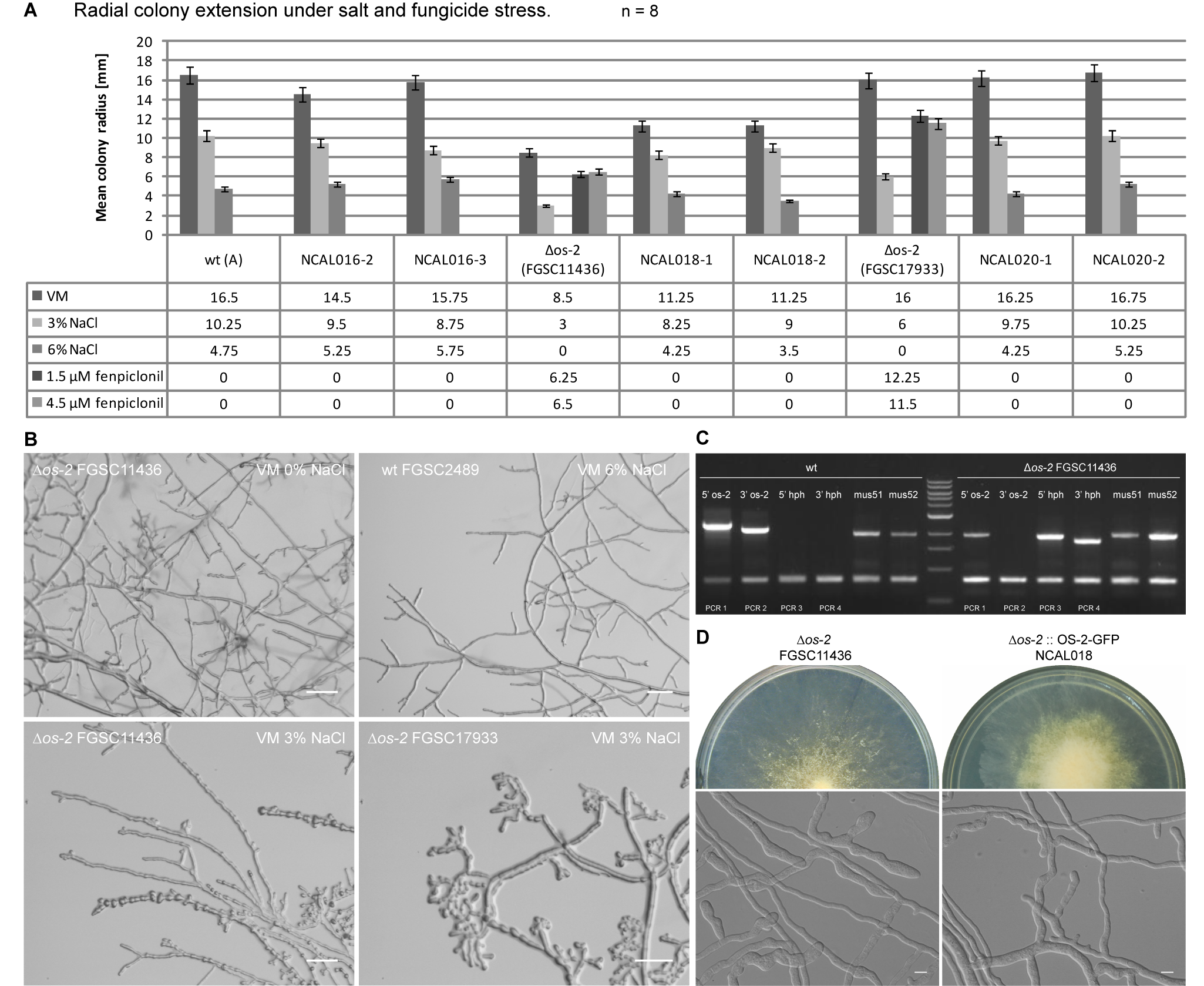

Supplement: Figure S4 — Phenotypic and genotypic characterization of Δ os-2 FGSC11436. (A) Differences in colony development, measured as radial colony extension, under salt and fungicide stress were compared between wild type, Δos-2 FGSC11436, Δos-2 FGSC17933 and corresponding genetically complemented transformants NCAL018 and NCAL020, respectively. The wild type was able to grow in the presence of 6% w/v NaCl in the medium, but unable to grow in the presence of the fungicide fenpiclonil (1.5 and 4.5 µM). This behavior was not altered through ectopic expression of OS-2-GFP in the wild type-transformant strains NCAL016-1 and -2. Due to its hyphal fusion defect, colony development of Δos-2 strain FGSC11436 was slower on the control medium. In addition, this strain showed increased sensitivity to salt stress and was unable to grow on medium supplemented with 6% NaCl. However, it was resistant to fenpiclonil. Osmosensitivity was rescued through ectopic expression of OS-2-GFP in the FGSC11436 transformants NCAL018-2 and -3, and its sensitivity to fenpiclonil was restored. With respect to osmosensitivity and fenpiclonil resistance, Δos-2 strain FGSC17933 and its corresponding OS-2-GFP transformants NCAL020-1 and -2, respectively, showed identical characteristics. (B) Normal hyphal morphology at the colony periphery of Δos-2 FGSC11436 under non-stress conditions and in the wild type in the presence of 6% salt. In the presence of 3% salt, both Δos-2 mutants show swollen hyphae and irregular growth pattern. Scale bars, 100 µm. (C) Multiplex PCR confirming that the native os-2 locus has been correctly exchanged for the hph-knock-out cassette. The 5′ os-2 signal is of the wrong size and thus most likely an unspecific product. Alternatively, this signal might indicate an unintended genetic alteration at the 5′-flank of the os-2 locus. Indicated PCRs 1-4 refer to the PCR set up shown in Figure S2. (D) Genetic complementation of Δos-2 FGSC11436 with os-2-gfp did not rescue the central-conidiation pheno [file pone.0042565.s004.tif]
