# Supplementary material for: Importance of MAP Kinases during Protoperithecial Morphogenesis in Neurospora crassa
Source: PLoS One. 2012 Aug 10;7(8):e42565. doi: 10.1371/journal.pone.0042565 (PMC3416862; doi:10.1371/journal.pone.0042565)
Supplement: Table S1 — Oligonucleotides used for PCR-genotyping. Please refer to Figures S1 and S2 to deduce primer positions and pairing. (DOCX) [file pone.0042565.s005.docx]

**Table S1 Primers used for PCR-genotyping.**

| **Primer name** | **5'-3' Oligonucleotide sequence** |
| --- | --- |
| nrc1_300_fw | GTCTTCTGGATGGCTCCTGA |
| 3f_nrc1 | GTTGCTACTCATCTGTGAGG |
| 3r_nrc1 | CTACCTACCGAGCTTCTTCC |
| mek2_300_fw | AGGTGGACCGTCTGTACATC |
| 3f_mek2 | GTCTCTGTCAAATGGGTAGC |
| 3r_mek2 | CTCACTTTCACATCGACTGC |
| mak2_300_fw | CTACTATGGCATCAAGTCGC |
| 3f_mak2 | GGTCTGATACGCTCTTATGG |
| 3r_mak2 | GTGGTGTAAGTTGCCTATCC |
| mik1_300_fw | GGTTGGGGCGATTTACAAGA |
| 3f_mik1 | CAGTCAGGGTTTACCATAGG |
| 3r_mik1 | AGAAGAGCCTCAAAGCTAGG |
| mek1_300_fw | CATATACCATCACCTCGGAC |
| 3f_mek1 | CCACCCCCTTAATATCAACC |
| 3r_mek1 | TGGTAGGTAAGGTAGCTTGG |
| mak1_300_fw | CGTATCAGCGTTGAGGATGC |
| 3f_mak1 | GGTCTTCAATGACAGAGTGG |
| 3r_mak1 | GGGTCATCAAGTACATCTCG |
| os2_5p200_fw | AGCGCATTAACCGTTTCGAG |
| os2_1000_rv | GTCGCAGAGCCTTCAGCAGAG |
| os2_500_fw | CCAGACGATGTCATCAACACG |
| os2_3p300_rv | GTGATCCGGAGATAGTGTGGGTATAAAC |
| os2_ORF_fw | GGAACTACCTTTGAGATCACCTCGAG |
| os2_ORF_rv | GGCTTCGACGTTGTGATAGTCCA |
| os4_5p200_fw | GTGATCACCACCTAGTAAGGCCTCC |
| os4_1000_rv | GGTCATGAGTTCCCTACTGGACG |
| os4_500_fw | GCTCACCGTGATATCAAGCCAG |
| os4_3p300_rv | GCAGCTAACAGGGTACTTGACAGG |
| os4_ORF_fw | CTACTTTGACGCTTACGGCTCC |
| os4_ORF_rv | GTCGTACAGTTGGTGGAGCATTTG |
| os5_5p200_fw | CCAAGGAGTACCGTGACCTGC |
| os5_1000_rv | CGTGGAAGCTTGGGCTTACTATG |
| os5_500_fw | GGTCTCACCATTATCGAGTGTGC |
| os5_3p300_rv | GATGCGCAACCATTTTCACC |
| os5_ORF_fw | ATCAACAGCGGATGGGCATG |
| os5_ORF_rv | GATCCATGGGTGCATCAGCAAC |
| mus51_fw | CACATCACACCATTTGACAGTC |
| mus51_rv | GATTCGAAGTGTTTTCTTGCCCA |
| mus52_fw | CATGCGCTACATCTGGGACA |
| mus52_rv | CATGCGCTACATCTGGGACA |
| mus51_2kb_fw | GTCATAGTGCTCCCAGGTCACA |
| mus51_2kb_rv | TGAATGCTCTGGTGTTCTGAAGA |
| mus52_2kb_fw | GGAAGCCACAGTCTATGTCATC |
| mus52_2kb_rv | CGCGTTTGCCTTGTACTTTTG |
| hph_800_fw | GAGAGCCTGACCTATTGCATCTC |
| hph_300_rv | TACCCGCAGGACATATCCA |
| hph_test_fw | GGAGGTCAACACATCAATGC |
| actin_58_fw | GACGACATGGAGAAGATTTGG |
| actin_58_rv | CTAGCCTATACTGCCATCTC |
| actin_70_fw | CCGCGAGGTGCTCTTGCTTGAGACG |
| actin_70_rv | GACCTGACCGTCGGGAAGCTCGTAG |
